# Digital Versus In-Person Physical Therapy in Adults With Musculoskeletal Conditions: Retrospective Matched-Cohort Analysis of Surgery and Low-Value Surgical Rates

**DOI:** 10.2196/82573

**Published:** 2025-12-17

**Authors:** Beatriz Domingues, Ana P Pereira, Akshat Pradhan, Catarina Zidde, Dora Janela, Carolina Marramaque, Virgílio Bento, Vijay Yanamadala, Steven P Cohen, Luke Belz, Kevin Wang, Fernando Dias Correia, Fabíola Costa

**Affiliations:** 1 Clinical Research & Dev Sword Health New York, NY United States; 2 Department of Surgery Frank H Netter School of Medicine, Quinnipiac University Hamden, CT United States; 3 Department of Neurosurgery Hartford Healthcare Medical Group Westport, CT United States; 4 Anesthesiology Department Northwestern University Feinberg School of Medicine Chicago, IL United States; 5 Departments of Anesthesiology and Physical Medicine and Rehabilitation and Anesthesiology Walter Reed National Military Medical Center, Uniformed Services University of the Health Sciences Bethesda, MD United States

**Keywords:** eHealth, low-value care, pain, surgery rate, telerehabilitation

## Abstract

**Background:**

Musculoskeletal (MSK) disorders are leading causes of disability worldwide, with clinical guidelines recommending physical therapy–based interventions. Digital MSK programs offer an alternative to address logistical and socioeconomic barriers to regular in-person care. However, evidence comparing surgical use between digital and in-person physical therapy remains limited, particularly for low-value procedures.

**Objective:**

This study aimed to evaluate the 12-month incidence of MSK surgery and low-value surgical procedures among participants initiating a multimodal Digital Care Program (DCP) versus a matched-cohort initiating in-person physical therapy.

**Methods:**

Retrospective, matched-cohort study, using exact and propensity matching, with a Health Insurance Portability and Accountability Act (HIPAA)–deidentified US nationwide merged claims dataset (July 2022-February 2025). Eligible adults had spine, knee, hip, or shoulder conditions, ≥24 months uninterrupted health insurance coverage to an employer-sponsored DCP, and no MSK surgery in the prior year. The intervention group (IG) participated in a DCP combining exercise, education, and cognitive behavioral therapy, with real-time biofeedback and remote physical therapist oversight. The comparator group (CG) initiated in-person physical therapy, identified from a third-party claims database, using relevant MSK *ICD-10* (*International Statistical Classification of Diseases, Tenth Revision*) codes as primary diagnosis. The primary outcome was the incidence of any MSK surgery within 12 months; the secondary outcome was the incidence of low-value surgery based on Choosing Wisely–aligned definitions. Cohort characteristics were compared using *t* test and chi-square test. Risk ratios (RRs) were calculated overall and by pain site, age group, and Social Deprivation Index.

**Results:**

In a matched cohort of 4190 individuals, predominantly middle-aged (~52 years old) women (1335/2095, 63.7%) with spinal pain (1123/2095, 53.6%), participation in the digital program was linked to a 58% (95% CI 49-66) lower relative risk of surgery at 12 months compared to those initiating in-person physical therapy (RR 0.42, 95% CI 0.34-0.52; E-value=4.19 [lower CI 3.29]). For surgeries categorized as low-value, IG was associated with 82% (95% CI 68-90) lower relative risk (RR 0.17, 95% CI 0.09-0.31; E-value=11.24 [lower CI 5.91]). Overall MSK surgical trends were consistent across pain sites, with greatest relative differences for knee (IG: 40/414 9.7% vs CG: 122/414, 29.5%; RR 0.26; 95% CI 0.17-0.38) followed by hip (19/203, 9.4% vs 42/203, 20.7%; RR 0.40; 95% CI 0.22-0.71). Lower surgery incidences in the IG (overall and low-value) were found across all socioeconomic and age strata.

**Conclusions:**

This real-world study demonstrated, for the first time, that participation in a digital MSK program was associated with substantially lower incidences of both overall and low-value surgeries compared to those who opted for in-person physical therapy among commercially-insured adults. These findings suggest that digital MSK programs can mitigate access barriers, promote adherence to guideline-concordant care, and reduce unnecessary procedures, including among underserved populations.

## Introduction

Musculoskeletal (MSK) disorders are among the most widespread health problems, being a leading contributor to years lived with disability worldwide [1-4]. In the United States, roughly half of adults live with an MSK condition [2,5], whose economic burden reaches an estimated US $980 billion annually, impacting patients, payers, and society as a whole [6].

MSK surgery rates have risen over the past decades in the United States. For example, incidences of elective lumbar spinal fusion rose markedly between 2002 and 2014, with an average annual increase of about 12% [4,7,8]. Similarly, shoulder arthroplasties increased substantially between 2011 and 2017, showing an average annual increase of approximately 13% [9]. More recently, from 2013 to 2022, total knee and hip arthroplasties continued to expand, rising by roughly 6%-8% per year [10,11]. These upward trajectories have raised significant concerns about overtreatment and the rising prevalence of low-value surgical procedures—defined as those offering no net clinical benefit in certain scenarios, especially when nonsurgical care has not been exhausted [12-18]. Such procedures may expose patients to unnecessary risks and prolonged recovery, without providing better outcomes than nonoperative interventions in several conditions [4,19-29].

Previous studies have reported that guideline-concordant physical therapy can promote better clinical outcomes (as greater functional improvement [30,31]), reduce health care resources use (eg, 28% lower risk of receiving advanced imaging [32]) and downstream MSK surgery (eg, 53% lower risk of receiving lumbar surgery [32]), with clinical guidelines uniformly endorsing physical therapy approaches, including exercise, education, and behavior change, as first-line care for most MSK presentations [33-38]. Yet, real-world uptake remains suboptimal [39,40], reflecting systemic barriers. The number of physical therapists varies greatly from state to state in the United States, ranging from 35 to 119 therapists per 100,000 individuals in 2021 [41]. Moreover, the distribution of clinicians is uneven across regions, leaving rural areas dependent on telehealth or traveling providers and limiting consistent access to regular in-person care, often delaying or preventing guideline-concordant treatment [42-47]. Additionally, modern lifestyles [43,45] and limited health literacy [46,48] may shape patients’ perspectives of surgery as a quicker or more definitive solution, even when it poses greater risks and limited benefits [19-22].

Digital care programs have emerged as a transformative strategy to improve adherence to evidence-based care, given their convenience [49-53]. By enabling easier access to care, it has the potential to curb avoidable surgeries [4,20,32,54,55]. We have previously shown, through randomized controlled trials, that a multimodal digital program—combining exercise, education, and cognitive behavioral therapy—can improve pain, function, and mental health, as effectively as in-person rehabilitation [56,57].

Despite growing evidence supporting the clinical effectiveness of digital MSK programs [50,52,58], there is limited evidence of their impact on surgical use, particularly regarding the reduction of low-value procedures. To address this gap, this study aims to compare 1-year incidences of MSK surgery among patients with spine, knee, hip, or shoulder conditions that initiated a Digital Care Program (DCP) and those initiating in-person physical therapy (primary aim), and to assess differences in the proportion of low-value surgeries between groups (secondary aim), in a commercially insured population.

## Methods

### Study Design

This was a retrospective matched cohort study comparing surgery incidences during 12 months between patients in a DCP (intervention group [IG]) to patients eligible for the DCP who opted for in-person physical therapy (comparator group [CG]) among a population of individuals enrolled in employer-sponsored commercial insurance plans that offered the DCP as a benefit. The comparison cohort was obtained using Health Insurance Portability and Accountability Act (HIPAA)–compliant, deidentified medical claims data sourced from a large third-party platform (PurpleLab) across the US health care claims spanned July 2022 to February 2025. Reporting of this study followed the RECORD (REporting of studies Conducted using Observational Routinely-collected Data) guidelines (Table S1 in Multimedia Appendix 1) [59].

### Study Population

Adults (≥18 years) who had an MSK-related index event from July 2023 to February 2024, residing in the United States (including all states) and with conditions related to spine, knee, hip, or shoulder, were included only if they had ≥12 months of uninterrupted health insurance coverage before and after their individual index event date. The MSK index event corresponded either to the date of enrollment in the DCP for the IG (obtained from DCP data collected through an observational clinical trial, refer to “Data Sources”) or to the date of the in-person MSK physical therapy evaluation visit in the CG (identified in claims, refer to “Comparator Group”). Targeted MSK conditions, reflecting conditions addressable by the DCP (including joint and soft tissue conditions, such as tendinopathies and arthropathies, affecting the spine, knee, hip, or shoulder), were identified through claims with relevant *ICD-10* (*International Statistical Classification of Diseases, Tenth Revision*) diagnostic codes (Table S2 in Multimedia Appendix 1). To ensure comparability and interpretability of outcomes, patients were required to have attended at least 1 physical therapy treatment session and have at least 1 MSK-coded medical claim in the 12 months preceding their index date. This criterion (1) established a preindex baseline for MSK care-seeking, use, and spending; (2) enabled matching and covariate adjustment using preindex characteristics; and (3) reduced misclassification of isolated or incidental presentations as index episodes. All patients with any MSK surgery in the 12-month preindex period (identified in medical claims through CPT [Current Procedural Terminology] and HCPCS [Healthcare Common Procedure Coding System] codes) were excluded. Additional exclusion criteria included patients with cancer, dementia, or conditions impairing the ability to follow motor instructions, pregnancy or childbirth (identified through *ICD-10* codes; Table S3 in Multimedia Appendix 1), and those with outlier total annual medical expenses detected through claims data (>US $500,000) during the full study period. These criteria were applied consistently to both the intervention (DCP) and comparison (in-person physical therapy) groups.

### Intervention Group

The DCP, a health benefit offered through 140 employers at no cost to patients, consisted of exercise, education, and cognitive behavioral therapy [56,57,60]. Enrollment was self-initiated by patients through a secure website, where a structured onboarding form was completed with demographics, clinical information, and red flags screening. Upon onboarding, each patient was assigned a Doctor of Physical Therapy who conducted a synchronous videocall assessment, in which eligibility was confirmed. The physical therapist subsequently prescribed and asynchronously monitored an individualized, guideline-based care plan [33-35,61]. Care was delivered via a US Food and Drug Administration (FDA)–listed Class II medical device, consisting of a tablet with motion-tracking technology for real-time audiovisual biofeedback during exercise. A companion smartphone app enabled 2-way communication and access to educational content, and interactive cognitive behavioral therapy modules [62,63].

### Comparator Group

The CG included individuals drawn from the eligible claims database who never enrolled in the DCP and had a claim with an MSK-related *ICD-10* code (Table S2 in Multimedia Appendix 1) as the primary diagnosis, categorized under physical therapy evaluation according to the Restructured BETOS Classification System (RBCS) [64] (CPT codes: 97161, 97162, 97163, or 97164), during the study period.

### Data Sources

Claims data for both groups were obtained from PurpleLab (schematic flow depicted in Figure S1 in Multimedia Appendix 1; summary of PurpleLab data source structure and data elements available are described in Text S1). A list of all available variables per group can be found in Table S4 in Multimedia Appendix 1, encompassing: (1) deidentified patient demographics (age, gender, geographic region, and Social Deprivation Index [SDI]); and (2) administrative medical claims data. Medical claims data are generated in the United States when health care providers submit standardized billing information to payers. These claims include fields such as diagnosis (*ICD-10* codes), health care procedure codes (CPT and HCPCS), service dates, and place of service.

Data obtained from Sword Health included longitudinal clinical data and engagement metrics of patients in the IG. Given the retrospective nature of the study, clinical data were not available for individuals in the CG, as they did not enroll in the DCP. Nevertheless, CG engagement was assessed via claims.

Before analysis, claims data preparation procedures were applied to ensure accuracy and consistency of code classifications and service groupings, following recommendations (Text S2 in Multimedia Appendix 1).

### Matching

Patients in the IG and CG were matched through both exact and propensity-matched scoring (Table 1; MatchIt package in R [version 4.2.2; R Foundation for Statistical Computing]), using 1:1 nearest-neighbor matching without replacement. A broad range of variables, ranging from demographic characteristics to MSK condition severity proxies, were applied. Propensity scores were estimated using a generalized linear model that included the covariates listed under “Propensity” (Table 1). Variable-specific calipers were used to restrict the allowable difference in propensity scores for matching. Balance between matched groups was assessed using standardized mean differences, with values of <0.10 considered indicative of acceptable balance (Figure S2 in Multimedia Appendix 1).

**Table 1:** Variables used for patients matching between the intervention group (DCP) and the comparison group (in-person physical therapy) in a retrospective claims-based matched-cohort analysis of patients with musculoskeletal conditions in the United States (July 2022-February 2025), specified separately for each applied method (ie, propensity score and exact matching).

Variables						Type			Description			Rationale for inclusion
**Exact matching**												
	**Age, sex, and geographic region**										To account for differences in care access, cost structures, and treatment patterns.	
			Age (years)	Categorical			18-29; 30-39; 40-49; 50-59; 60-69; 70-79					
			Sex	Categorical			Male; Female					
			Geographic region	Categorical			Midwest; Northeast; South; West					
	**MSK^a^** **pain site and acuity**										To reflect condition type and severity, influencing care needs.	
			MSK pain site	Categorical			Primary body region affected by the MSK condition, grouped by *ICD-10*^b^ codes for low back, neck, knee, hip or shoulder)					
			Acuity	Categorical			Short-term recent; Short-term ongoing; Chronic. Short-term recent corresponds to patients with a single claim for the pain site of the index event up to its date; Short-term ongoing refers to patients with at least 2 related claims, with the first occurring less than 12 weeks prior to the index date; Chronic reflects episodes lasting more than 12 weeks with 2 or more related claims [65]					
	**Number of MSK conditions in the past 3 months,** **physical therapy visits, and imaging visits**										To account for prior MSK-related health care use, ongoing care pathways, and patient preferences in treatment-seeking behavior.	
			Number of MSK conditions in the past 3 months	Categorical			1; 2+					
			Physical therapy visits	Categorical			0; 1+					
			Imaging visits	Categorical			0; 1+					
**Propensity**												
		Elixhauser Comorbidity Index [66,67]			Continuous			Score calculated based on *ICD-10* codes			To control for overall health status and chronic disease complexity.	
		Preindex total MSK-related health care spending			Continuous			Total health care costs related to MSK conditions. All costs were standardized using the Centers for Medicare & Medicaid Services Fee Schedules and expressed in 2024 US dollars			To reflect care intensity and pain burden, which are predictive of future health care use.	
		Social Deprivation Index [63,68]			Categorical			0-20; 21-40; 41-60; 61-80; 81-100			To reflect the impact of social determinants of health.	

^a^MSK: musculoskeletal.

^b^ICD-10: International Statistical Classification of Diseases, Tenth Revision.

### Outcomes

#### Comparing Digital to In-Person Physical Therapy

The primary outcome of this study was the incidence of MSK surgery in the 12 months after initiating the digital MSK program (IG) or in-person physical therapy (CG). CPT and HCPCS codes were used to identify MSK surgeries in the medical claims (Table S5 in Multimedia Appendix 1). Visits occurring on the same service date for a beneficiary were treated as a single event. Analyses were run for the entire cohort and stratified by pain area (spine, knee, hip, or shoulder).

#### Evaluating Potential Low-Value Care

The study evaluated low-value surgeries previously defined [12,14-16,18], and aligned with Choosing Wisely recommendations [69]. It included clinically diverse, claims-based indicators of low-value care (the coding algorithm for each surgery indicator and the underlying evidence base are detailed in Table S6 in Multimedia Appendix 1). To ensure alignment with robust evidence, these indicators were reviewed against recommendations from the best available research and current clinical practice guidelines [34-38]. The targeted surgeries, included spinal fusion, knee procedures (meniscectomy for degenerative joint disease and arthroplasty for osteoarthritis), hip arthroplasty for osteoarthritis, and shoulder procedures (rotator cuff repair, replacement for osteoarthritis, arthroscopic release or manipulation for frozen shoulder, arthroscopy or open distal clavicle resection for impingement syndrome or arthritis, acromioplasty or acromiotomy for rotator cuff disorders, and labral repair). In order to identify if a given surgery was low value or not, a stringent set of inclusion and exclusion criteria had to be met (Table S6 in Multimedia Appendix 1). Particularly, in the case of conditions for which conservative should be first-line (knee arthroplasty for knee osteoarthritis, hip arthroplasty for hip osteoarthritis, rotator cuff repair for partial tears, shoulder replacement for osteoarthritis, arthroscopic release or manipulation under anesthesia for frozen shoulder, shoulder arthroscopy or distal clavicle resection for rotator cuff disorders or arthritis, and shoulder labral repair for labral tears), these would only be considered low value if no physical therapy was used throughout the 6 months period before surgery date. As such, in the IG group, the 6-month preperiod was screened for both DCP session logs and physical therapy medical claims, while in the CG group, the screening was run through physical therapy medical claims (Table S6 in Multimedia Appendix 1). The 6-month criterion was aligned with international benchmarks on orthopedic surgical waiting times, as the reported median waiting time was approximately 172 days for hip replacement and 119 days for knee replacement [70].

#### Engagement in Digital and In-Person Physical Therapy

Engagement was evaluated over the 12-month postindex period for patients receiving either digital or in-person physical therapy, based on physical therapy visit claims in the CG and claims and logged participation in the IG.

#### Assessing Digital Intervention Clinical Outcomes

Leveraging the availability of clinical outcomes for the IG: Numerical Pain Rating Scale (NPRS), Work Productivity and Activity Impairment (WPAI) scale, and satisfaction data, these were analyzed to support the clinical interpretation of findings. Clinical outcomes were collected at baseline, 9th, 19th, and 24th sessions in the IG (Table S7 in Multimedia Appendix 1).

### Statistical Analysis

Baseline characteristics of matched cohorts were descriptively reported and compared through independent *t* test and chi-square test, with a statistical significance level of .05. Surgery incidences were computed as the number of patients who underwent surgery divided by the total sample size per group over the 12-month follow-up period. MSK surgery counts within 12 months postindex date were assessed. To evaluate the association between DCP participation and surgical use (including low-value procedures), risk ratios (RRs) were computed directly from the observed incidence proportions, and 95% CIs were estimated using the Wald method. E-values were calculated to assess the robustness of the observed associations to potential unmeasured confounding, using the formula proposed by VanderWeele and Ding [71,72]. For protective associations (RR<1), the reciprocal of the RR was used. E-values were computed for both the point estimate and the CI limit closest to the null. Absolute risk reduction and number needed to treat (NNT) were calculated to quantify the magnitude of the effect. For metrics stratified by pain sites (spine, knee, hip, and shoulder), pain site assignment was defined as the primary diagnosis code (*ICD-10*) associated with the claim corresponding to the index event after matching and duplicate removal, ensuring that each patient was assigned to a single pain site group and included in the study only once.

To explore potential heterogeneity in treatment effects, subgroup analyses were conducted by SDI categories (C1: 0-25; C2: 26-50; C3: 51-75; C4: 76-100) and by age group (18-39, 40-59, ≥60 years). Within each stratum, surgery incidences and RRs were estimated.

Clinical outcomes among IG patients were evaluated under an intention-to-treat framework using latent-basis growth analysis (LBGA) [73], a longitudinal modeling technique that accommodates nonlinear change, handles missing data via full-information maximum likelihood, and provides model fit [74,75]. A robust sandwich estimator was used for SEs. Analyses were also performed for patients with baseline clinically-relevant scores: WPAI-overall>0, WPAI-activities>0. Pain response rate was calculated using 30% or 2-points improvement in NPRS as the Minimal Clinically Important Change (MCIC) [76].

### Sensitivity Analyses

To assess the robustness of findings to unmeasured confounding and model misspecification, we conducted a sensitivity analysis using augmented inverse probability weighting (AIPW). The AIPW analysis included the same covariates used in the main matching procedure (Table 1). Propensity scores were estimated for all eligible individuals (before matching) using logistic regression, and these scores were applied within the AIPW framework to obtain doubly robust estimates of the average treatment effect. The outcome model used logistic regression for the binary end points (any surgery and low-value surgery). AIPW combines outcome regression with inverse-probability weighting to produce valid inference if either model is correctly specified. Results are reported as adjusted RRs (95% CIs).

As an additional bias assessment, a negative control analysis comparing rates of non-MSK elective procedures (cataract surgery and colonoscopy) between groups was conducted to detect residual confounding or systematic error, as these outcomes should not plausibly be influenced by MSK care.

To evaluate the robustness of the 6-month preoperative observation window criterion to define low-value procedures (refer to “Evaluating Potential Low-Value Care”), a sensitivity analysis applying alternative look-back windows of 3, 9, and 12 months prior to surgery was conducted. For each window, the total number of low-value surgeries, both overall and stratified by pain site, was calculated.

All statistical analyses were conducted using R (version 4.2.2; R Foundation for Statistical Computing) and commercially available software (SPSS version 22; IBM Corp). This significance level was set at .05 for all 2-sided hypothesis tests.

### Ethical Considerations

This research was conducted in accordance with the Declaration of Helsinki and all applicable ethical guidelines and regulations. The IG (digital MSK group) was created under a prospective observational study that was reviewed and approved by the Advarra Institutional Review Board (Pro00063337) with electronic informed consent provided by all included participants, which included authorization for future secondary analyses. For the retrospective claims-based analysis, all claims data were HIPAA-deidentified and tokenized by PurpleLab using Datavant Tokens prior to transfer to the study team. Sword Health applied the same Datavant tokenization process to its member eligibility and enrollment data, enabling secure linkage between datasets while maintaining full deidentification. Both data streams remained anonymous throughout analysis, and the study team had no access to any identifiable information. Data privacy compliance and reidentification risk mitigation were verified by Promeed Inc [77] through expert review to ensure adherence to HIPAA standards. Importantly, no individual can be identified from the manuscript or underlying data. Since no identifiable private information was accessed by the researchers, the regulatory definition of human subjects research [78-80] was not met; therefore, institutional review board review or additional informed consent was not required. Participants were not offered any form of compensation.

## Results

### Baseline Characteristics

**Figure 1:**
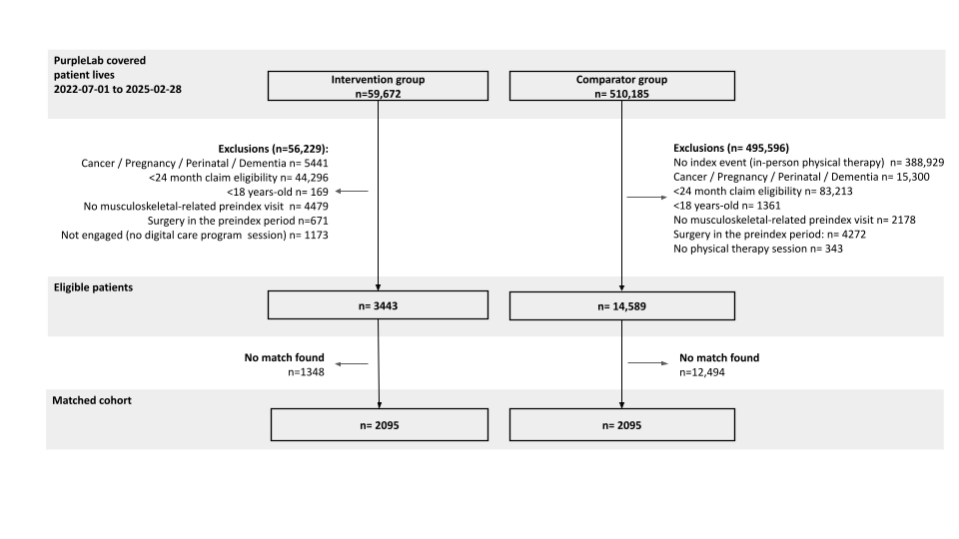
Study cohorts and flowchart.

From 569,857 individuals included in the dataset, the eligible population included 3443 individuals in the IG and 14,589 in the CG (study cohort flowchart in Figure 1). The matched cohort yielded a final analytical sample of 4190 participants.

Baseline demographic and clinical characteristics were balanced between the IG and CG (N=2095 each) after matching, with no statistically significant differences observed (see exact *P* values in Table 2; Figure S2 in Multimedia Appendix 1; Table 2). Both groups were predominantly female (1335/2095, 63.79%), and their mean ages differed by –0.3 years (SMD=–0.02, 95% CI –0.09 to 0.04). Preindex MSK-related costs mean difference was US $78.3 (SMD=0.06, 95% CI –0.00 to 0.12). Baseline geographic region and pain site distributions were equivalent between cohorts, as a reflection of the exact matching procedure. Most patients resided in the Midwest US region (1751/2095, 83.6%), consistent with the geographic distribution of individuals eligible for the DCP. Spine pain was the most common site (1123/2095, 53.6%), followed by knee (414/2095, 19.8%), shoulder (355/2095, 16.9%), and hip (203/2095, 9.7%; Table 2).

**Table 2:** Baseline characteristics of the intervention group (Digital Care Program) and the comparison group (in-person physical therapy) in a retrospective claims-based matched-cohort analysis of patients with musculoskeletal conditions in the United States (July 2022-February 2025).

Baseline characteristics				Intervention group (N=2095)		Comparator group (N=2095)		*P* value	
**Demographic characteristics**									
	Sex, n (%)								>.99
		Female	1335 (63.7)		1335 (63.7)				
		Male	760 (36.3)		760 (36.3)				
	Age (years), mean (SD)			52.3 (10.5)		52.0 (10.9)		.44	
	**Age categories (years), n (%)**								>.99
		18-29	75 (3.6)		75 (3.6)				
		30-39	190 (9.1)		190 (9.1)				
		40-49	465 (22.2)		465 (22.2)				
		50-59	786 (37.5)		786 (37.5)				
		60-69	559 (26.7)		559 (26.7)				
		70-79	20 (1.0)		20 (1.0)				
	**Geographic US region, n (%)**								>.99
		Midwest	1751 (83.6)		1751 (83.6)				
		South	233 (11.1)		233 (11.1)				
		West	97 (4.6)		97 (4.6)				
		Northeast	14 (0.7)		14 (0.7)				
	**Social Deprivation Index^a^** **, n (%)**								.09
		C1: 0-25	822 (39.2)		851 (40.6)				
		C2: 26-50	567 (27.1)		570 (27.2)				
		C3: 51-75	414 (19.8)		436 (20.8)				
		C4: 76-100	292 (13.9)		238 (11.4)				
**Clinical data**									
	**Pain site, n (%)**								>.99
		Spine	1123 (53.6)		1123 (53.6)				
		Knee	414 (19.8)		414 (19.8)				
		Hip	203 (9.7)		203 (9.7)				
		Shoulder	355 (16.9)		355 (16.9)				
	**Acuity^b^** **, n (%)**								>.99
		Short-term recent	462 (22.1)		462 (22.2)				
		Short-term ongoing	957 (45.6)		957 (45.7)				
		Chronic	676 (32.3)		676 (32.3)				
	Number of patients with concurrent MSK^c^ conditions 3 months preindex (yes/no), n (%)			612 (29.2)		612 (29.2)		>.99	
	**Weighted Elixhauser Comorbidity Index^d^** **, n (%)**								.65
		<0	696 (33.2)		692 (33.1)				
		0	1194 (56.8)		1217 (58.1)				
		1-4	137 (6.5)		118 (5.6)				
		≥5	68 (3.2)		68 (3.2)				
**Preindex care**									
	**MSK-related health care use, n (%)**								
		Physical therapy (yes/no)	897 (42.8)		897 (42.8)		>.99		
		Imaging (yes/no)	1377 (65.6)		1377 (65.6)		>.99		
	MSK-related costs (US $), mean (SD)			1166.4 (1326.5)		1244.6 (1338.1)		.06	
	TCOC^e^ (US $), mean (SD)			5805.8 (11,320.5)		6330.8 (12,404.7)		.11	

^a^Higher Social deprivation index values correspond to higher level of social deprivation [63,68,81].

^b^Short-term recent corresponds to patients with a single claim for the pain site of the index event up to its date; Short-term ongoing refers to patients with at least 2 related claims, with the first occurring less than 12 weeks prior to the index date; chronic reflects episodes lasting more than 12 weeks with 2 or more related claims [65].

^c^MSK: musculoskeletal.

^d^Higher Elixhauser scores indicate higher comorbidity burden.

^e^TCOC: total all-cause costs of care.

### Comparing Surgeries in Digital and In-Person Physical Therapy

Over 12 months, patients in the digital MSK program had 58.3% lower surgery incidence than those who received in-person physical therapy: 5.5% (115/2095) underwent MSK surgery in the IG versus 13.2% (276/2095) in the CG (Table 3). This corresponds to a 58% (95% CI 49-66) lower relative risk of surgery (RR 0.42, 95% CI 0.34-0.52; Table 3; E-value=4.19 [lower CI 3.29]), consistent with results from the AIPW sensitivity analysis (refer to “Sensitivity Analyses” subsection). Estimated absolute risk reduction was 7.7 percentage points (95% CI 6.0-9.4), meaning that for every 13 patients undergoing the DCP, one surgery is prevented within 1 year (NNT=13; 95% CI 11-17).

Surgery incidences were consistent and significantly lower in the IG than the CG across all pain sites. The largest difference was observed for knee surgeries (40/414, 9.7% vs 122/414, 29.5%; RR 0.26, 95% CI 0.17-0.38), followed by hip (19/203, 9.4% vs 42/203, 20.7%; RR 0.40, 95% CI 0.22-0.71), shoulder (19/355, 5.4% vs 38/355, 10.7%; RR 0.47, 95% CI 0.27-0.84), and spine (37/1123, 3.3% vs 74/1123, 6.6%; RR 0.48, 95% CI 0.32-0.72; Table 3).

**Figure 2:**
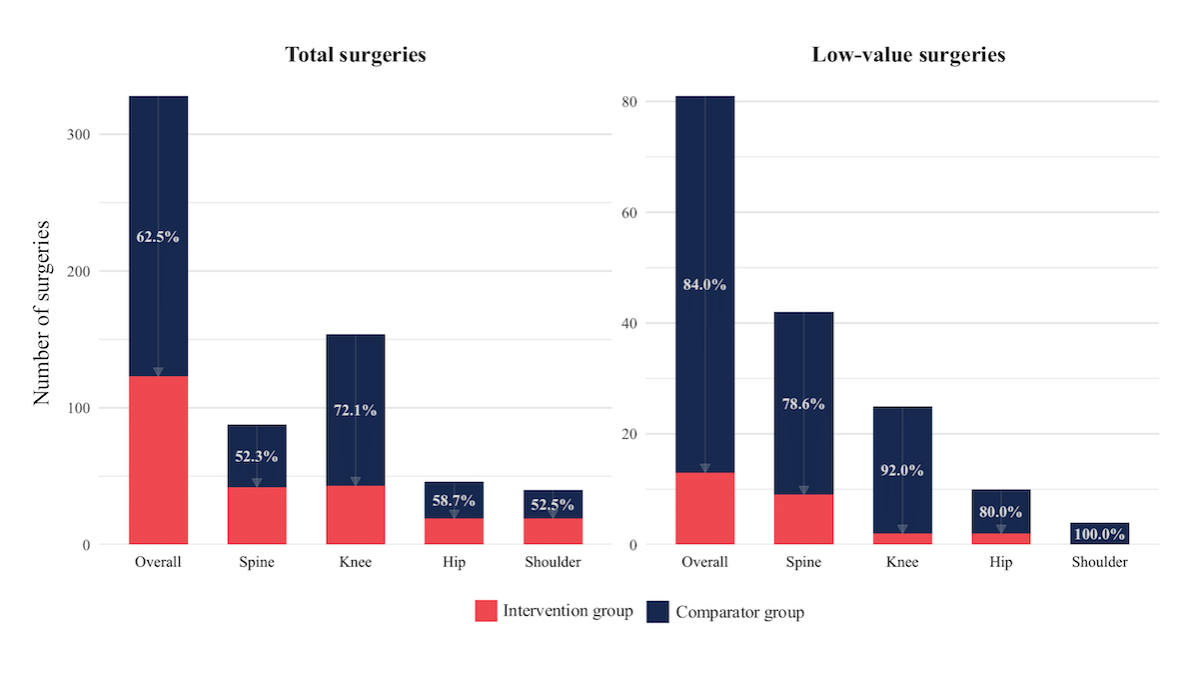
Postindex surgery use at 1 year (proportions) between the intervention group (Digital Care Program) and the comparison group (in-person physical therapy) in a retrospective claims-based matched-cohort analysis of patients with musculoskeletal conditions in the United States (July 2022-February 2025). Percentages inside the bars and the arrow represent the relative surgery differences obtained in the intervention group compared to the comparison group. Results are shown overall and stratified by pain site.

Among patients who underwent surgery in the postindex period, 7.0% (8/115) of the IG had 2 or more surgeries, versus 15.9% (44/276) of the CG (*P*=.03). Overall, the IG had 62.5% (205/328) fewer surgeries compared to the CG, with the most substantial differences occurring in the knee (111/154, 72.1%) and hip (27/46, 58.7%) regions (Figure 2; Table S8 in Multimedia Appendix 1). Figure 2 presents the relative difference, in percentage, in surgery counts between the IG and CG; arrows denote the direction and magnitude of reduction.

**Table 3:** Comparison of 1-year incidence of surgeries and low-value surgeries between the intervention group (Digital Care Program) and the comparison group (in-person physical therapy) in a retrospective claims-based matched-cohort analysis of patients with musculoskeletal conditions in the United States (July 2022-February 2025). Results are presented overall and stratified by pain site.^a^

Pain site and outcome			IG^b^		CG^c^		RR^d^ (95% CI)
**Overall (N=2095), n (%)**							
	Any surgery	115 (5.5)		276 (13.2)		0.42 (0.34-0.52)	
	Low-value surgery	13 (0.6)		74 (3.5)		0.17 (0.09-0.31)	
**Spine (n=1123), n (%)**							
	Any surgery	37 (3.3)		74 (6.6)		0.48 (0.32-0.72)	
	Low-value surgery	9 (0.8)		37 (3.3)		0.24 (0.11-0.49)	
**Knee (n=414), n (%)**							
	Any surgery	40 (9.7)		122 (29.5)		0.26 (0.17-0.38)	
	Low-value surgery	2 (0.5)		23 (5.5)		0.08 (0.02-0.35)	
**Hip (n=203), n (%)**							
	Any surgery	19 (9.4)		42 (20.7)		0.40 (0.22-0.71)	
	Low-value surgery	2 (1.0)		10 (4.9)		0.19 (0.04-0.89)	
**Shoulder (n=355), n (%)**							
	Any surgery	19 (5.4)		38 (10.7)		0.47 (0.27-0.84)	
	Low-value surgery	0 (0.0)		4 (1.1)		—^e^	

^a^n represents the number of patients in the cohort who had surgery, and N represents the total number of patients in that cohort.

^b^IG: Intervention group.

^c^CG: Comparator group.

^d^RR: risk ratio.

^e^Not available.

### Comparing Low-Value Surgeries in Digital and in-Person Physical Therapy

Incidents of surgeries identified as low-value among patients in the DCP were lower by 82.4% in comparison to CG (Table 3). Overall, 0.6% (13/2095) of patients in the DCP underwent a low-value surgery, compared to 3.5% (74/2095) in the CG, corresponding to an 83% (95% CI 68-90) lower risk of surgery (RR 0.17; 95% CI 0.09-0.31; Table 3; E-value=11.24 [lower CI 5.91]), which was consistent with AIPW results (refer to “Sensitivity Analyses” subsection). This represents an absolute risk reduction of 2.9 percentage points (95% CI 2.1-3.8), corresponding to an NNT of 34 (95% CI 27-49) to prevent one low-value surgery.

Similar to the overall trend, low-value surgery incidences across pain sites were consistently lower in the IG than CG for spine (0.8%, 9/1123 vs 3.3%, 37/1123, RR 0.24; 95% CI 0.11-0.49), knee (0.5%, 2/414 vs 5.5%, 23/414; RR 0.08; 95% CI 0.02-0.35), and hip (1.0%, 2/203 vs 4.9%, 10/203; RR 0.19; 95% CI 0.04-0.89). In the shoulder cohort, no patient in the IG had low-value surgeries, compared to 4 in the CG (Table 3).

Among patients who underwent low-value surgery, 5 individuals in the CG had 2 or more surgeries, compared to zero individuals in the IG (*P*>.99). Overall, IG had 84.0% (68/81) fewer low-value surgeries compared to CG, with the largest differences observed in the shoulder (4/4, 100.0%) and knee (23/25, 92.0%) cohorts (Figure 2; Table S8 in Multimedia Appendix 1).

### Sensitivity Analyses

Results from the AIPW analysis were consistent with the matched analysis, showing an overall RR 0.45 (95% CI 0.35-0.57) and a low-value RR 0.19 (95% CI 0.10-0.35; Table S9 in Multimedia Appendix 1), confirming the robustness of the findings.

Negative control analyses comparing rates of cataract and colonoscopy procedures (outcomes not plausibly affected by MSK care) showed no significant differences between groups before or after the intervention, supporting the absence of residual systematic bias (see exact *P* values in Table S10 in Multimedia Appendix 1).

Sensitivity analyses varying the preoperative observation window (3, 9, and 12 months) yielded consistent results, showing similarly lower counts of surgeries in the IG (Table S11 in Multimedia Appendix 1).

### Subgroup Analyses by SDI and Age

Surgery incidences in the IG were consistently lower across all social deprivation levels, with relative differences starting around 50% among more deprived groups (SDI C1-C4: 49.2%-70.0%; RRs ranged from 0.30, 95% CI 0.19-0.47 to 0.51, 95% CI 0.37-0.70; Table S12 and Figure S3 in Multimedia Appendix 1). Moreover, a significantly lower likelihood of receiving low-value care was observed in the IG than CG across SDI categories, with differences ranging from 84.9% to 88.4% in the most deprived categories (SDI C2-C4, RRs ranging from 0.12, 95% CI 0.01-0.94 to 0.15, 95% CI 0.05-0.50), compared with a difference of 75.0% in the least deprived group (SDI C1, RR: 0.25, 95% CI 0.11-0.57; Table S12 in Multimedia Appendix 1).

Age-stratified analyses also showed a significant benefit toward for the IG at a similar level across age groups (18-39 years: RR 0.42, 95% CI 0.19-0.94; 40-59 years: RR 0.42, 95% CI 0.31-0.56; ≥60 years: RR 0.42, 95% CI 0.30-0.57), with relative differences in low-value surgeries all exceeding 70.4% (18-39 years: no low-value surgeries observed in the IG; 40-59 years: RR 0.13, 95% CI 0.05-0.32; ≥60 years: RR 0.30, 95% CI 0.14-0.65; Table S12 in Multimedia Appendix 1; Figure S3 in Multimedia Appendix 1).

### Engagement in Digital and In-Person Physical Therapy

The IG performed 47,526 physical therapy sessions, including 42,067 digital sessions, over the 12-month postindex period, corresponding to a mean of 22.7 (SD 31.6, median 14, IQR 9-25) sessions. All patients in the CG initiated in-person physical therapy care, collectively completed 19,047 sessions corresponding on average to 9.2 (SD 9.3, median 6, IQR 3-12) sessions over the 12-month postindex period.

### Digital Intervention Clinical Outcomes

To support the clinical interpretation of the surgery incidence in the IG, clinical outcomes were evaluated through the available DCP data. Patients experienced a significant reduction in pain by program-end (mean change: –2.22 points, 95% CI –2.35 to –2.08; *P*<.001), with 63.8% (1036/1626) achieving clinically meaningful relief, alongside high satisfaction (mean 8.7/10, SD 1.8). Further information is available in Table S13 in Multimedia Appendix 1.

## Discussion

### Principal Results

This study compared 1-year incidences of MSK surgeries between patients with spine, knee, hip, or shoulder conditions who initiated a DCP versus those who initiated in-person physical therapy. Following our primary aim, this study demonstrated that participation in the DCP was associated with a significantly lower incidence of surgery at 1 year. Regarding our secondary aim, the DCP group also demonstrated a substantially lower incidence of surgeries classified as low-value based on clinical guidelines. The robustness of these results was supported by several bias assessment sensitivity analyses, including AIPW, negative control analysis, and E-value estimation. In this large, real-world, matched cohort of 4190 commercially insured adults, surgery incidence was 58% lower overall and 82% lower for low-value procedures in the DCP group compared with in-person physical therapy. Results were consistent across pain sites, socioeconomic, and age strata. These findings underscore the potential of digitally delivered multimodal MSK interventions to reduce low-value surgical use at the population level.

### Comparison With Prior Work

Evidence on the role of digital programs in avoiding surgery is limited [82,83]. Herein, we used a nationwide sample, diverse in terms of demographics, socioeconomic status, and pain sites, comparable to the US population with MSK conditions [2,40], to evaluate the impact of a digital care solution in promoting access and adherence to guideline-concordant care.

Extending prior work that contrasted physical therapy with the absence or delay of care [32,39], this study underscores the potential impact of alternative delivery modality on downstream outcomes. Alongside lower surgical incidences, the percentage of patients who underwent multiple surgeries was lower in the IG. Literature suggests that undergoing an MSK surgery can increase the likelihood of additional surgical interventions [84]. The DCP cohort experienced a 58% (95% CI 49-66) reduction in the risk of undergoing MSK surgery, consistent across pain sites, in alignment with prior literature [82,83], with the largest relative benefit in knee-related conditions and the smallest for spinal disorders. The smaller difference in spine surgery rates is likely attributable to less standardized and more variable selection of surgical candidates than for other orthopedic surgeries, and more subjective outcomes than many other surgeries [85-87].

From available data, it was observed that the total number of sessions completed in the IG exceeded the observed in the CG, whose average engagement is aligned with prior claims studies for in-person therapy [88]. While session duration may differ between digital and in-person settings, the marked engagement gap highlights the greater convenience and compliance associated with remote interventions. These results suggest that the DCP is capable of facilitating more sustained engagement, potentially being an inherent component of its real-world effectiveness. The obstacles associated with in-person visits, including scheduling, transportation, and caregiving responsibilities, may explain high rates of no-shows [42,46] or dropouts [43,56,57]. Nevertheless, the mechanistic assessment of the observed results is outside the scope of this study, given its retrospective observational design, which precludes causal inference. Higher adherence has been shown to produce better outcomes [89], which in turn reduces the chances of care escalation [20,49]. In a previous study, 31% of participants initially willing to consider joint replacement no longer considered surgery after completing a multimodal digital osteoarthritis program, with this shift strongly associated with reduced pain and improved function at follow-up [90]. The findings of this study are aligned with the literature as patients in the DCP experienced clinically meaningful improvements in pain and function consistent with benchmarks observed in prior randomized trials [55,56].

Low-value MSK procedures—such as meniscectomy in degenerative knee disease or spinal fusion in the absence of instability or red flags—are costly, carry substantial procedural risks, and often do not improve outcomes compared to conservative care [19-22]. In this study, patients in the DCP had 83% (95% CI 68-90) lower risk of low-value procedures compared to CG, with E-values deeming these estimations robust to unmeasured confounding. This trend remained stable even when addressing different prior conservative care evaluation windows. Among those seeking care for spine pain, a high proportion of those who followed a surgical path underwent low-value care, in accordance with literature [14,91], which was less frequent among the IG. Reducing low-value surgery aligns with national value-based care priorities [69,92], aiming to reduce the overuse of medical procedures that yield little benefit or cause harm—a problem that has persisted despite recent policy initiatives [12,13,15]. Scalable digital programs may represent a practical and sustainable solution to achieve this goal, particularly among vulnerable populations.

The subgroup analyses revealed consistently lower MSK surgery incidences in the IG across social deprivation and age groups. Studies have shown the impact social determinants of health have on both care use and surgical outcomes [93-95]. Herein, relatively low-value surgery incidence differences among those more deprived underscore the potential of the DCP to promote equitable outcomes for diverse populations. The results suggest that when persistent barriers to access and care compliance are overcome, patients from more disadvantaged backgrounds can achieve benefits comparable to those from less deprived settings [63].

These findings highlight the potential of digital care models as modifiers of surgical decision-making. By combining convenience, enhanced adherence, and delivery of evidence-based, patient-centered care, digital programs can empower individuals to actively manage their condition—fostering confidence and autonomy that could delay or avert surgical interventions. These implications are relevant for payers and health systems seeking scalable strategies to reduce unnecessary procedures and promote recovery—particularly in remote or underserved populations where access to in-person MSK treatment remains limited. This study offers a framework for evaluating digital health interventions as tools to increase care value.

### Limitations and Future Directions

This retrospective study has inherent limitations. Although we applied exact and propensity score matching and several sensitivity analyses, including AIPW and negative control analysis, to mitigate bias, residual confounding from unmeasured factors cannot be excluded. Albeit claims-based analyses have the advantage of objectively capturing in a real-world context the health care use through standardized coding at large scale, these analyses are dependent on coding accuracy and lack clinical granularities (disease severity, imaging findings, time from symptoms onset to physical therapy initiation, patient preferences, and provider decision-making). Specifically for low-value care, claims may not capture all clinically appropriate indications—a common limitation in low-value care studies [14,15,18]. To mitigate this risk, we have applied a standardized data validation protocol, used the most specific service definitions available, used validated criteria from prior research, incorporated conditions severity, health care use and patients’ preferences in treatment-seeking behavior proxies for matching, conducted a thorough literature review and consulted a panel of expert clinicians to minimize misclassification.

The predominance of participants residing in the Midwest may limit the generalizability of our findings, as regional differences in MSK surgery practice patterns [96] and access to employer-sponsored DCP may not reflect the national landscape. Consistent findings across subgroups, sensitivity analyses, and analytic approaches increase confidence, but the 12-month follow-up cannot distinguish surgeries averted from those deferred beyond the observation window. The spine category included both low back and neck cases; however, we acknowledge that the overall pathways of care differ between these regions, including surgical indications and physical therapy regimens.

Future studies should assess the mechanisms underlying the observed results, namely through randomized controlled trials. Qualitative studies exploring patient decision-making may shed light on behavioral mechanisms contributing to lower surgery rates. Additionally, future studies should assess the generalizability of findings across different geographic populations, insurance types, and care settings, as well as longer evaluation timeframes. Cost-effectiveness analyses are also warranted, given the potentially substantial implications for payer budgets and value-based purchasing.

### Conclusions

This study demonstrated, for the first time, within a large, real-world, commercially insured cohort, that providing access to digital MSK care was associated with substantially lower incidences of surgical procedures, including those deemed as low-value. Effects were consistent across pain sites, age groups, and socioeconomic strata.

These findings have important real-world implications, showing that increasing access and convenience to physical therapy through the form of digital programs can promote treatment compliance and lower health care use, namely preventable surgical care. By supporting more equitable and value-based care delivery, such programs may represent a scalable and alternative model for MSK and resource management in routine clinical practice.

## Appendix

Multimedia Appendix 1 Additional figures, tables, and text.

## Abbreviations

AIPW: Augmented Inverse Probability Weighting
CG: comparator group
CPT: Current Procedural Terminology
DCP: Digital Care Program
FDA: US Food and Drug Administration
HCPCS: Healthcare Common Procedure Coding System
HIPAA: Health Insurance Portability and Accountability Act
ICD-10: International Statistical Classification of Diseases, Tenth Revision
IG: intervention group
LBGA: latent-basis growth analysis
MCIC: Minimal Clinically Important Change
MSK: musculoskeletal
NNT: number needed to treat
NPRS: Numerical Pain Rating Scale
RBCS: Restructured BETOS Classification System
RECORD: REporting of studies Conducted using Observational Routinely-collected Data
RR: risk ratio
SDI: Social Deprivation Index
WPAI: Work Productivity and Activity Impairment

## Data Availability

The datasets generated or analyzed during this study are not publicly available due to contractual obligations to PurpleLab but deidentified data may be available from the corresponding author on reasonable request.
