# Supplementary material for: Digital Versus In-Person Physical Therapy in Adults With Musculoskeletal Conditions: Retrospective Matched-Cohort Analysis of Surgery and Low-Value Surgical Rates
Source: J Med Internet Res. 2025 Dec 17;27:e82573. doi: 10.2196/82573 (PMC12756660; doi:10.2196/82573)
Supplement: Multimedia Appendix 1 [file jmir_v27i1e82573_app1.docx]

**Supplementary Material**

# Table S1. RECORD (REporting of studies Conducted using Observational Routinely-collected health Data) checklist [1].

|  | **Item No.** | **STROBE items** | **Location in manuscript where items are reported** | **RECORD items** | **Location in manuscript where items are reported** |
| --- | --- | --- | --- | --- | --- |
| **Title and abstract** | | | | | |
|  | 1 | (a) Indicate the study’s design with a commonly used term in the title or the abstract (b) Provide in the abstract an informative and balanced summary of what was done and what was found | (a) Pages 1 & 2 (b) Pages 2-4 | RECORD 1.1: The type of data used should be specified in the title or abstract. When possible, the name of the databases used should be included.  RECORD 1.2: If applicable, the geographic region and timeframe within which the study took place should be reported in the title or abstract.  RECORD 1.3: If linkage between databases was conducted for the study, this should be clearly stated in the title or abstract. | 1.1: Pages 2 & 3     1.2: Page 2  1.3: Page 2 |
| **Introduction** | | | | | |
| Background rationale | 2 | Explain the scientific background and rationale for the investigation being reported | Pages 5 & 6 |  |  |
| Objectives | 3 | State specific objectives, including any prespecified hypotheses | Page 6 |  |  |
| **Methods** | | | | | |
| Study Design | 4 | Present key elements of study design early in the paper | Pages 6 & 7 |  |  |
| Setting | 5 | Describe the setting, locations, and relevant dates, including periods of recruitment, exposure, follow-up, and data collection | Page 7 & 8 |  |  |
| Participants | 6 | *(a) Cohort study* - Give the eligibility criteria, and the sources and methods of selection of participants. Describe methods of follow-up  *Case-control study* - Give the eligibility criteria, and the sources and methods of case ascertainment and control selection. Give the rationale for the choice of cases and controls  *Cross-sectional study* - Give the eligibility criteria, and the sources and methods of selection of participants  *(b) Cohort study* - For matched studies, give matching criteria and number of exposed and unexposed  *Case-control study* - For matched studies, give matching criteria and the number of controls per case | (a) Pages 7-9  (b) Pages 9-11, Supplementary Figure S2, Table 1 | RECORD 6.1: The methods of study population selection (such as codes or algorithms used to identify subjects) should be listed in detail. If this is not possible, an explanation should be provided.  RECORD 6.2: Any validation studies of the codes or algorithms used to select the population should be referenced. If validation was conducted for this study and not published elsewhere, detailed methods and results should be provided.  RECORD 6.3: If the study involved linkage of databases, consider use of a flow diagram or other graphical display to demonstrate the data linkage process, including the number of individuals with linked data at each stage. | 6.1: Pages 7 & 8, Supplementary Table S2, Supplementary Table S3, Supplementary Figure S1  6.2: Supplementary Material S2  6.3: Supplementary Figure S1 |
| Variables | 7 | Clearly define all outcomes, exposures, predictors, potential confounders, and effect modifiers. Give diagnostic criteria, if applicable. | Pages 11-13 | RECORD 7.1: A complete list of codes and algorithms used to classify exposures, outcomes, confounders, and effect modifiers should be provided. If these cannot be reported, an explanation should be provided. | Pages 11-13, Supplementary Table S2, Supplementary Table S5, Supplementary Table S6 & Supplementary Table S7 |
| Data sources/ measurement | 8 | For each variable of interest, give sources of data and details of methods of assessment (measurement).  Describe comparability of assessment methods if there is more than one group | Pages 9, 11-13, 14-16,  Supplementary Figure S1, Supplementary Material S1 & Supplementary Table S4 |  |  |
| Bias | 9 | Describe any efforts to address potential sources of bias | Pages 9-11, 14-16, Supplementary Figure S2 & Table 1 |  |  |
| Study size | 10 | Explain how the study size was arrived at | Page 16, Figure 1 |  |  |
| Quantitative variables | 11 | Explain how quantitative variables were handled in the analyses. If applicable, describe which groupings were chosen, and why | Pages 11-13, 14, 15 & Table 1 |  |  |
| Statistical methods | 12 | (a) Describe all statistical methods, including those used to control for confounding  (b) Describe any methods used to examine subgroups and interactions  (c) Explain how missing data were addressed  (d) *Cohort study* - If applicable, explain how loss to follow-up was addressed  *Case-control study* - If applicable, explain how matching of cases and controls was addressed  *Cross-sectional study* - If applicable, describe analytical methods taking account of sampling strategy  (e) Describe any sensitivity analyses | (a) Pages 14-16  (b) Pages 14-16  (c) Page 15  (d) Not applicable  (e) Pages 14-16 & Supplementary Table S9 |  |  |
| Data access and cleaning methods |  | .. |  | RECORD 12.1: Authors should describe the extent to which the investigators had access to the database population used to create the study population.  RECORD 12.2: Authors should provide information on the data cleaning methods used in the study. | 12.1: Pages 13 & 14  12.2: Page 9 & Supplementary Material S2 |
| Linkage |  | .. |  | RECORD 12.3: State whether the study included person-level, institutional-level, or other data linkage across two or more databases. The methods of linkage and methods of linkage quality evaluation should be provided. | Page 9, 13, 14, Supplementary Figure S1 & Supplementary Table S4 |
| **Results** | | | | | |
| Participants | 13 | (a) Report the numbers of individuals at each stage of the study (*e.g.*, numbers potentially eligible, examined for eligibility, confirmed eligible, included in the study, completing follow-up, and analysed)  (b) Give reasons for non-participation at each stage.  (c) Consider use of a flow diagram | (a) Figure 1, Page 16  (b) Figure 1  (c) Figure 1 | RECORD 13.1: Describe in detail the selection of the persons included in the study (*i.e.,* study population selection) including filtering based on data quality, data availability and linkage. The selection of included persons can be described in the text and/or by means of the study flow diagram. | Pages 16-20, Figure 1 &  Table 2 |
| Descriptive data | 14 | (a) Give characteristics of study participants (*e.g.*, demographic, clinical, social) and information on exposures and potential confounders  (b) Indicate the number of participants with missing data for each variable of interest  (c) *Cohort study* - summarise follow-up time (*e.g.*, average and total amount) | (a) Pages 16-20 & Table 2  (b) Supplementary Table S13  (c) Pages 20-25, Table 3, Figure 2, Supplementary Table S8, Supplementary Table S12 & Supplementary Table S13 |  |  |
| Outcome data | 15 | *Cohort study* - Report numbers of outcome events or summary measures over time  *Case-control study* - Report numbers in each exposure category, or summary measures of exposure  *Cross-sectional study* - Report numbers of outcome events or summary measures | Pages 20-25, Table 3, Figure 2, Supplementary Table S8, Supplementary Table S12 & Supplementary Table S13 |  |  |
| Main results | 16 | (a) Give unadjusted estimates and, if applicable, confounder-adjusted estimates and their precision (e.g., 95% confidence interval). Make clear which confounders were adjusted for and why they were included  (b) Report category boundaries when continuous variables were categorized  (c) If relevant, consider translating estimates of relative risk into absolute risk for a meaningful time period | (a) Pages 20-24, Table 3, Figure 2, Supplementary Table S8, Supplementary Table S12, Supplementary Table S13 & Supplementary Figure S3  (b) Page 23, Supplementary Table S12, Supplementary Figure S3  (c) Pages 20-24, Table 3 & Supplementary Table S12, Supplementary Figure S3 |  |  |
| Other analyses | 17 | Report other analyses done—e.g., analyses of subgroups and interactions, and sensitivity analyses | Pages 23-24, Supplementary Table S9, Supplementary Table S10, Supplementary Table S11, Supplementary Table S12 & Supplementary Figure S3 |  |  |
| **Discussion** | | | | | |
| Key results | 18 | Summarise key results with reference to study objectives | Pages 24 & 25 |  |  |
| Limitations | 19 | Discuss limitations of the study, taking into account sources of potential bias or imprecision. Discuss both direction and magnitude of any potential bias | Pages 27-29 | RECORD 19.1: Discuss the implications of using data that were not created or collected to answer the specific research question(s). Include discussion of misclassification bias, unmeasured confounding, missing data, and changing eligibility over time, as they pertain to the study being reported. | Pages 27-29 |
| Interpretation | 20 | Give a cautious overall interpretation of results considering objectives, limitations, multiplicity of analyses, results from similar studies, and other relevant evidence | Pages 25-29 |  |  |
| Generalisability | 21 | Discuss the generalisability (external validity) of the study results | Pages 28-29 |  |  |
| **Other Information** | | | | | |
| Funding | 22 | Give the source of funding and the role of the funders for the present study and, if applicable, for the original study on which the present article is based | Page 30 |  |  |
| Accessibility of protocol, raw data, and programming code |  | .. | Page 30 | RECORD 22.1: Authors should provide information on how to access any supplemental information such as the study protocol, raw data, or programming code. | Page 30 |

*Checklist is protected under Creative Commons Attribution ([CC BY](http://creativecommons.org/licenses/by/4.0/)) license.

# Table S2. ICD-10 primary diagnosis codes considered to identify the MSK medical claims.

| **ICD-10 Major Category** | **Diagnostic Category** | **Full ICD-10 Codes** |
| --- | --- | --- |
| G54 | Nerve root and plexus disorders | G540, G541, G542, G543, G544 |
| G56 | Mononeuropathies of upper limb | G5640, G5641, G5642, G5643, G5680, G5681, G5682, G5683, G5690, G5691, G5692, G5693 |
| G57 | Mononeuropathies of lower limb | G57, G5700, G5701, G5702, G5703, G5710, G5711, G5712, G5713, G5720, G5721, G5722, G5723, G5730, G5731, G5732, G5733, G5740, G5741, G5742, G5743, G5750, G5751, G5752, G5753, G5760, G5761, G5762, G5763, G5770, G5771, G5772, G5773, G5780, G5781, G5782, G5783, G5790, G5791, G5792, G5793 |
| M12 | Other and unspecified arthropathy | M1230, M1231, M1235, M1236, M1238, M1239, M1240, M1241, M1245, M1246, M1248, M1249, M1250, M1251, M1255, M1256, M1258, M1259, M1280, M1281, M1285, M1286, M1288, M1289, M129 |
| M13 | Other arthritis | M1310, M1311, M1315, M1316, M1380, M1381, M1385, M1386, M1388, M1389 |
| M14 | Arthropathies in other diseases | M1460, M1461, M1465, M1466, M1468, M1469, M1480, M1481, M1485, M1486, M1488, M1489 |
| M15 | Polyarthrosis | M150, M153, M154, M158, M159 |
| M16 | Osteoarthritis of hip | M16, M160, M1610, M1611, M1612, M162, M1630, M1631, M1632, M164, M1650, M1651, M1652, M166, M167, M169 |
| M17 | Osteoarthritis of knee | M17, M170, M1710, M1711, M1712, M172, M1730, M1731, M1732, M174, M175, M179 |
| M19 | Other and unspecified osteoarthritis | M1901, M1909, M1911, M1919, M1921, M1929, M1990, M1991, M1992, M1993 |
| M21 | Other acquired deformities of limbs | M2100, M2105, M2106, M2110, M2115, M2116, M2120, M2121, M2125, M2126 |
| M22 | Disorder of patella | M22, M2200, M2201, M2202, M2210, M2211, M2212, M222X, M223X, M2240, M2241, M2242, M228X, M2290, M2291, M2292 |
| M23 | Internal derangement of knee | M23, M230, M2300, M2301, M2302, M2303, M2304, M2305, M2306, M232, M2320, M2321, M2322, M2323, M2324, M2325, M2326, M233, M2330, M2331, M2332, M2333, M2334, M2335, M2336, M234, M2340, M2341, M2342, M235, M2350, M2351, M2352, M236, M2360, M2361, M2362, M2363, M2364, M2367, M238, M238X, M239, M2390, M2391, M2392 |
| M24 | Other specific joint derangements | M2400, M2401, M2405, M2410, M2411, M2415, M2420, M2421, M2425, M2428, M2429, M2430, M2431, M2435, M2436, M2439, M2440, M2441, M2445, M2446, M2450, M2451, M2455, M2456, M247, M2480, M2481, M2485, M2489, M249 |
| M25 | Other joint disorder, not elsewhere classified | M2511, M2515, M2516, M2518, M2530, M2531, M2535, M2536, M2539, M2540, M2541, M2545, M2546, M2548, M2550, M2551, M2555, M2556, M2560, M2561, M2565, M2566, M2569, M2570, M2571, M2575, M2576, M2578, M2580, M2581, M2585, M2586, M259 |
| M40 | Kyphosis and lordosis | M40, M4000, M4003, M4004, M4005, M4010, M4012, M4013, M4014, M4015, M4020, M4029, M4030, M4035, M4036, M4037, M4040, M4045, M4046, M4047, M4050, M4055, M4056, M4057 |
| M41 | Scoliosis | M41, M4100, M4102, M4103, M4104, M4105, M4106, M4107, M4108, M4111, M4112, M4120, M4122, M4123, M4124, M4125, M4126, M4127, M4130, M4134, M4135, M4140, M4141, M4142, M4143, M4144, M4145, M4146, M4147, M4150, M4152, M4153, M4154, M4155, M4156, M4157, M4180, M4182, M4183, M4184, M4185, M4186, M4187 |
| M42 | Spinal osteochondrosis | M42, M4200, M4201, M4202, M4203, M4204, M4205, M4206, M4207, M4208, M4209, M4210, M4211, M4212, M4213, M4214, M4215, M4216, M4217, M4218, M4219, M429 |
| M43 | Other deforming dorsopathies | M43, M431, M4310, M4311, M4312, M4313, M4314, M4315, M4316, M4317, M4318, M4319, M432, M4320, M4321, M4322, M4323, M4324, M4325, M4326, M4327, M4328, M435, M435X, M438, M438X, M439 |
| M45 | Ankylosing spondylitis | M45, M450, M451, M452, M453, M454, M455, M456, M457, M458, M459 |
| M46 | Other inflammatory spondylopathies | M46, M460, M4600, M4601, M4602, M4603, M4604, M4605, M4606, M4607, M4608, M4609, M461, M468, M4680, M4681, M4682, M4683, M4684, M4685, M4686, M4687, M4688, M4689, M469, M4690, M4691, M4692, M4693, M4694, M4695, M4696, M4697, M4698, M4699 |
| M47 | Spondylosis | M47, M4720, M4721, M4722, M4723, M4724, M4725, M4726, M4727, M4728, M4781, M4789, M479 |
| M48 | Other spondylopathies | M4820, M4821, M4822, M4823, M4824, M4825, M4826, M4827, M4830, M4831, M4832, M4833, M4834, M4835, M4836, M4837, M4838, M488X, M489 |
| M49 | Spondylopathies in diseases classified elsewhere | M4980, M4981, M4982, M4983, M4984, M4985, M4986, M4987, M4988, M4989 |
| M50 | Cervical disc disorders | M5010, M5011, M5012, M5013, M5020, M5021, M5022, M5023, M5030, M5031, M5032, M5033, M5080, M5081, M5082, M5083, M5090, M5091, M5092, M5093 |
| M51 | Thoracic, thoracolumbar, and lumbosacral intervertebral disc disorders | M5114, M5115, M5116, M5117, M5124, M5125, M5126, M5127, M5134, M5135, M5136, M5137, M5144, M5145, M5146, M5147, M5184, M5185, M5186, M5187, M519 |
| M53 | Other and unspecified dorsopathies, not elsewhere classified | M530, M531, M532X, M533, M5380, M5381, M5382, M5383, M5384, M5385, M5386, M5387, M5388, M539 |
| M54 | Dorsalgia | M5410, M5411, M5412, M5413, M5414, M5415, M5416, M5417, M5418, M542, M5430, M5431, M5432, M5440, M5441, M5442, M545, M5450, M5451, M5459, M546, M549 |
| M62 | Other disorders of muscle | M6200, M6201, M6202, M6205, M6206, M6208, M6211, M6212, M6215, M6216, M6240, M6241, M6242, M6245, M6246, M6248, M6249, M6251, M6255, M6256, M6258, M6259, M6281, M6283, M6289, M629 |
| M65 | Synovitis and tenosynovitis | M6520, M6522, M6525, M6526, M6528, M6580, M6581, M6582, M6585, M6586, M6588, M659 |
| M66 | Spontaneous rupture of synovium and tendon | M6611, M6615, M6620, M6621, M6622, M6625, M6626, M6628, M6629, M6630, M6631, M6632, M6635, M6636, M6638, M6639, M6680, M6681, M6682, M6685, M6686, M6688, M6689, M669 |
| M67 | Other disorders of synovium and tendon | M6720, M6721, M6722, M6725, M6726, M6728, M6729, M6730, M6731, M6735, M6736, M6738, M6739, M6740, M6741, M6745, M6746, M6748, M6749, M6750, M6751, M6752, M6780, M6781, M6785, M6786, M6788, M6789, M6790, M6791, M6792, M6795, M6796, M6798 |
| M70 | Soft tissue disorders related to use, overuse and pressure | M7040, M7041, M7042, M7050, M7051, M7052, M7060, M7061, M7062, M7070, M7071, M7072, M7080, M7081, M7082, M7085, M7086, M7088, M7089, M7090, M7091, M7092, M7095, M7096, M7097, M7098, M7099 |
| M71 | Other bursopathies | M7120, M7121, M7122, M7130, M7131, M7135, M7138, M7139, M7140, M7145, M7146, M7148, M7149, M7150, M7155, M7156, M7158, M7180, M7181, M7185, M7186, M7188, M7189, M719 |
| M75 | Shoulder lesions | M7500, M7501, M7502, M7510, M7511, M7512, M7520, M7521, M7522, M7530, M7531, M7532, M7540, M7541, M7542, M7550, M7551, M7552, M7580, M7581, M7582, M7590, M7591, M7592 |
| M76 | Enthesopathies, lower limb, excluding foot | M7600, M7601, M7602, M7610, M7611, M7612, M7620, M7621, M7622, M7630, M7631, M7632, M7640, M7641, M7642, M7650, M7651, M7652, M7670, M7671, M7672, M7681, M7682, M7689, M769 |
| M77 | Other enthesopathies | M778, M779 |
| M79 | Other and unspecified soft tissue disorders, not elsewhere classified | M7912, M7918, M794, M7960, M7962, M7965, M7966 |
| M84 | Disorder of continuity of bone | M8430, M8431, M8432, M8435, M8436 |
| M93 | Other osteochondropathies | M9320, M9321, M9325, M9326, M9328, M9329, M9380, M9381, M9382, M9385, M9386, M9388, M9389 |
| M94 | Other disorders of cartilage | M9420, M9421, M9425, M9426, M9428, M9429, M9435, M948X |
| M95 | Other acquired deformities of musculoskeletal system and connective tissue | M953, M955 |
| M96 | Intraoperative and postprocedural complications and disorders of musculoskeletal system, not elsewhere classified | M961 |
| M99 | Biomechanical lesions, not elsewhere classified | M9901, M9902, M9903, M9904, M9905, M9906, M9911, M9912, M9913, M9914, M9915, M9916, M9961, M9962, M9963, M9964, M9965, M9966, M9967, M9969, M9970, M9971, M9972, M9973, M9974, M9975, M9976, M9977 |
| S13 | Dislocation and sprain of joints and ligaments at neck level | S134X, S138X, S139X |
| S16 | Injury of muscle, fascia and tendon at neck level | S161X, S168X, S169X |
| S30 | Superficial injury of abdomen, lower back, pelvis and external genitals | S300X |
| S33 | Dislocation and sprain of joints and ligaments of lumbar spine and pelvis | S335X, S336X, S338X, S339X |
| S34 | Injury of lumbar and sacral spinal cord and nerves at abdomen, lower back and pelvis level | S3421, S3422 |
| S39 | Other and unspecified injuries of abdomen, lower back, pelvis and external genitals | S3901, S3909, S3982, S3992 |
| S40 | Superficial injury of shoulder and upper arm | S4001, S4002 |
| S43 | Dislocation and sprain of joints and ligaments of shoulder girdle | S4330, S4340, S4341, S4342, S4343, S4349, S4350, S4351, S4352, S4380, S4381, S4382, S4390, S4391, S4392 |
| S44 | Injury of nerves at shoulder and upper arm level | S4400, S4401, S4402, S4410, S4411, S4412, S4420, S4421, S4422, S4430, S4431, S4432, S4440, S4441, S4442, S448X, S4490, S4491, S4492 |
| S46 | Injury of muscle, fascia and tendon at shoulder and upper arm level | S4600, S4601, S4609, S4610, S4611, S4619, S4620, S4621, S4629, S4630, S4631, S4639, S4680, S4681, S4689, S4690, S4691, S4699 |
| S70 | Superficial injury of hip and thigh | S7000, S7001, S7002, S7010, S7011, S7012 |
| S73 | Dislocation and sprain of joint and ligaments of hip | S7310, S7311, S7312, S7319 |
| S76 | Injury of muscle, fascia and tendon at hip and thigh level | S7600, S7601, S7609, S7610, S7611, S7619, S7620, S7621, S7629, S7630, S7631, S7639, S7680, S7681, S7682, S7689, S7690, S7691, S7699 |
| S83 | Fracture of lower leg, including ankle | S8320, S8321, S8322, S8323, S8324, S8325, S8326, S8327, S8328, S8330, S8331, S8332, S8340, S8341, S8342, S8350, S8351, S8352, S8360, S8361, S8362, S838X, S8390, S8391, S8392 |
| S86 | Injury of muscle, fascia and tendon at lower leg level | S8611, S8619, S8620, S8621, S8629, S8630, S8631, S8639, S8680, S8681, S8682, S8689, S8690, S8691, S8692, S8699 |
| S89 | Other and unspecified injuries of lower leg | S8980, S8981, S8982, S8990, S8991, S8992 |
| Z96 | Presence of other functional implants | Z9661, Z9664, Z9665 |

**Abbreviations:** ICD-10, International Classification of Diseases, 10th Revision.

# Table S3. Categorized ICD-10 Codes and CCSR groups for ICD-10-CM categories used to identify patients with conditions that warrant exclusion from the study.

| **Major Category** | **Diagnostic Category** | **Full Codes** |
| --- | --- | --- |
| NEO (CCSR 74) | Neoplasms | NEO001-NEO025, NEO028-NEO071 (except NEO073) |
| PNL (CCSR 13)  PRG (CCSR 30) | Perinatal period,  and Pregnancy, Childbirth and the Puerperium | PNL001-PNL013, PRG001-PRG030 |
| F0, F7, G2, G3, G7, R2 (ICD-10) | Dementia, Cognitive impairment or Intellectual disabilities | F01-F03, F70-F79, G20, G30, G71, R26 |

**Abbreviations:** CCSR, Clinical Classifications Software Refined; ICD-10, International Classification of Diseases, 10th Revision; ICD-10-CM, International Classification of Diseases, 10th Revision, Clinical Modification.

#

#

#

#

#
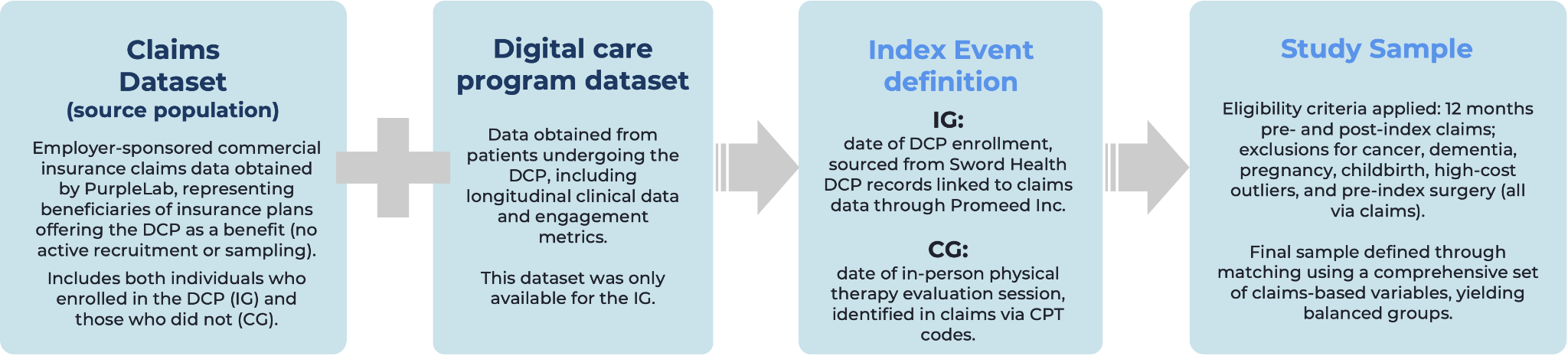


# Figure S1. Schematic representation of the data collection process and cohort definition for the intervention and comparison groups.

**Abbreviations:** CG, Comparator group; CPT, Current Procedural Terminology; DCP, Digital Care Program; IG, Intervention Group.

# Text S1. Brief description of PurpleLab data source.

PurpleLab is one of the largest claims data repositories in the US (covering millions of lives) and aggregates fully adjudicated, payer sourced claims via direct feeds and partnerships rather than purchasing a commercial database license. In practice, that means:

**Third-Party Administrators (TPAs) & Self-Insured Employers**

Clients deliver member eligibility & claims extracts (medical, pharmacy, ancillary) via secure SFTP or API. These feeds span both national administrators and large regional/local TPAs.

**National & Regional Carrier Partnerships**

For fully insured groups, PurpleLab ingests de-identified claims from major carriers under data-share agreements.

Key data elements available:

1. Enrollment files: demographics, eligibility history (coverage dates, plan tier, employer ID);

2. Medical claims: inpatient, outpatient, office, emergency room, imaging, surgery, ancillary; includes ICD-10, CPT/HCPCS, place-of-service, provider National Provider Identifier, billed/allowed/paid amounts;

3. Ancillary & lab: select lab orders/results, Durable Medical Equipment (DME), home health;

4. Longitudinal linkage via de-identified member & claim IDs.

# Table S4. Variables by Intervention and Comparator Groups With Data Sources

| **Variables** | **Sources** | |
| --- | --- | --- |
|  | **Intervention Group** | **Comparator Group** |
| **Demographic characteristics** | |  |
| Gender | PurpleLab claims data | |
| Age | PurpleLab claims data | |
| Geographic U.S. region | PurpleLab claims data | |
| Social Deprivation Index | PurpleLab claims data | |
| **Clinical data** |  |  |
| Index event date | DCP data | PurpleLab claims data |
| Index event condition | DCP data | PurpleLab claims data |
| Pain site | DCP data | PurpleLab claims data |
| Acuity | PurpleLab claims data | |
| Number of concurrent MSK conditions 3 months pre-index | PurpleLab claims data | |
| Weighed Elixhauser Comorbidity Index | PurpleLab claims data | |
| MSK surgery (visits and dates) | PurpleLab claims data | |
| MSK-related healthcare utilization (physical therapy visits, imaging visits) | PurpleLab claims data | |
| MSK-related costs | PurpleLab claims data | |
| Total costs of care | PurpleLab claims data | |
| Digital physical therapy sessions | DCP data | NA |
| **Clinical outcomes** |  |  |
| Pain | DCP data | NA |
| Work productivity and Daily Activities | DCP data | NA |
| Satisfaction | DCP data | NA |

**Abbreviations:** DCP, Digital Care Program; NA, not available; MSK, Musculoskeletal.

# Text S2. Claims Classification and Validation Procedures.

To ensure accurate and consistent classification of musculoskeletal (MSK)-related claims, we applied a standardized data validation protocol aligned with established recommendations for retrospective claims analyses [2, 3].

MSK-related diagnoses within the scope of physical therapy and addressable by the digital care program were identified using ICD-10 codes.

Service types (e.g., surgery, rehabilitation, imaging) were assigned using the Restructured BETOS Classification System, combined with CPT/HCPCS codes and place-of-service indicators, according to CMS conventions. Misclassified or clinically irrelevant codes were excluded, and consistency checks were performed to flag implausible sequences (e.g., surgery preceding diagnosis). All cost data were normalized using CMS Fee Schedules to reduce payer-level variability and ensure comparability across claims.

#

#


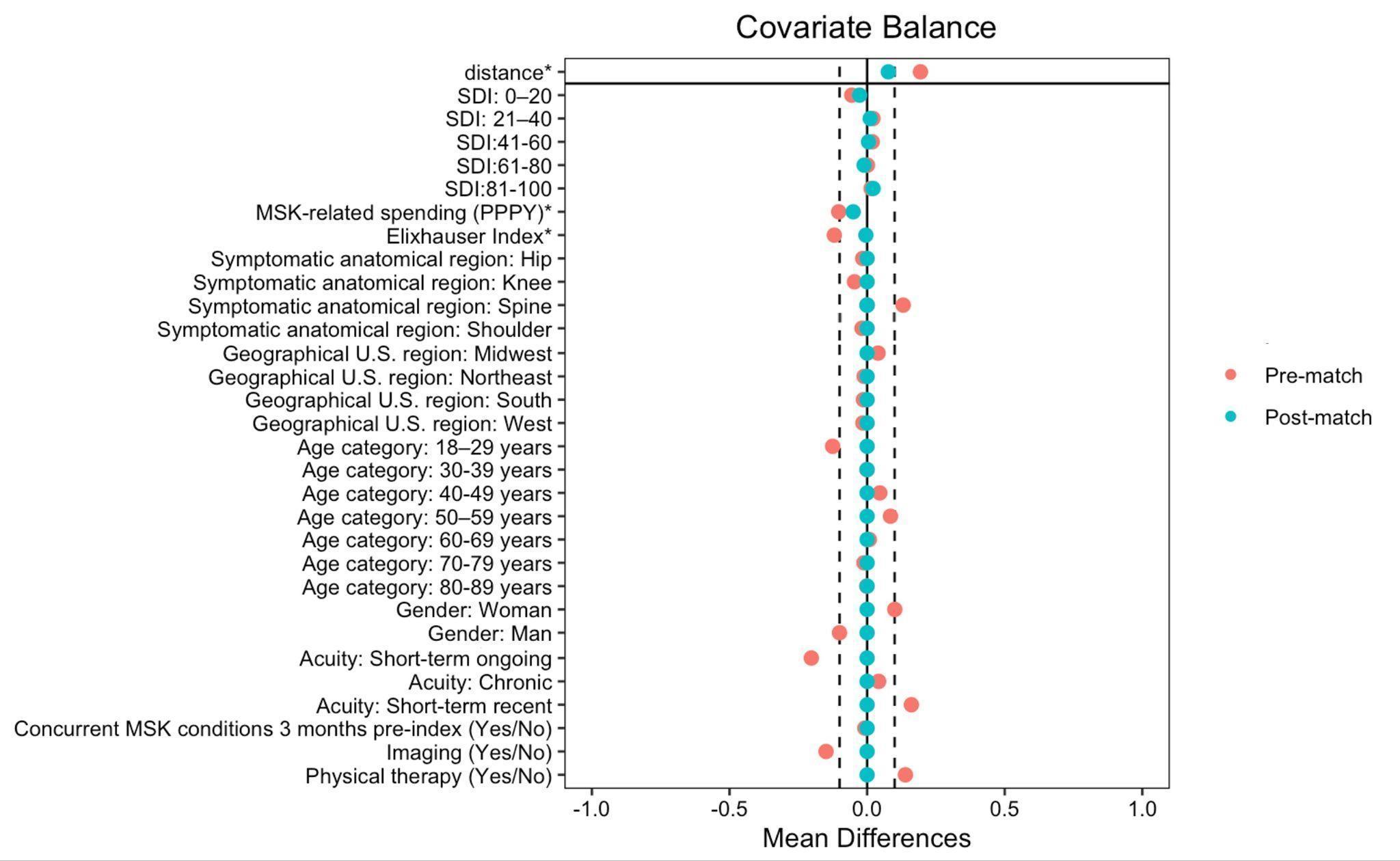


# Figure S2. Standardized mean differences between groups of the variables used for patients matching.

#

# Table S5. Procedure codes used to identify musculoskeletal surgeries by pain site.

| **Site** | **Procedure code** | **Description** |
| --- | --- | --- |
| Spine | 0163T | Tot Disc Arthrp Ant Appr Ea Addl Ntrspc Lumbar |
| Spine | 0656T | Ant Lumbar/Tlmbr Vrt Body Tethrg <7Vrt Seg |
| Spine | 11770 | Excision Pilonidal Cyst/Sinus Simple |
| Spine | 13100 | Repair Complex Trunk 1.1-2.5 Cm |
| Spine | 13101 | Repair Complex Trunk 2.6-7.5 Cm |
| Spine | 13102 | Repair Complex Trunk Each Additional 5 Cm/< |
| Spine | 14001 | Adjnt Tis Transfr/Rearrange Trunk 10.1-30.0 Sqcm |
| Spine | 15734 | Musc Myocutaneous/Fasciocutaneous Flap Trunk |
| Spine | 20650 | Insertion Wire/Pin W/Appl Skeletal Traction Spx |
| Spine | 20930 | Allograft For Spine Surgery Only Morselized |
| Spine | 20931 | Allograft For Spine Surgery Only Structural |
| Spine | 20936 | Autograft Spine Surgery Local From Same Incision |
| Spine | 20937 | Autograft Spine Surgery Morselized Sep Incision |
| Spine | 20938 | Autograft Spine Surgery Bicort/Tricort Sep Inc |
| Spine | 21600 | Excision Rib Partial |
| Spine | 21615 | Excision 1St &/Cervical Rib |
| Spine | 21616 | Excision 1St &/Cervical Rib W/Sympathectomy |
| Spine | 21700 | Division Scalenus Anticus W/O Rescj Cervical Rib |
| Spine | 21705 | Division Scalenus Anticus Resection Cervical Rib |
| Spine | 22100 | Prtl Exc Pst Vrt Intrnsc B1Y Les 1 Vrt Sgm Crv |
| Spine | 22102 | Prtl Exc Pst Vrt Intrnsc B1Y Les 1 Vrt Sgm Lmbr |
| Spine | 22110 | Prtl Exc Vrt Bdy B1Y Les W/O Spi Cord 1 Sgm Crv |
| Spine | 22116 | Prtl Exc Vrt Bdy B1Y Les W/O Spi Cord 1 Sgm Ea |
| Spine | 22206 | Osteotomy Spine Posterior 3 Column Thoracic |
| Spine | 22207 | Osteotomy Spine Posterior 3 Column Lumbar |
| Spine | 22210 | Osteotomy Spine Pst/Pstlat Appr 1 Vrt Sgm Crv |
| Spine | 22212 | Osteotomy Spine Pst/Pstlat Appr 1 Vrt Sgm Thrc |
| Spine | 22214 | Osteotomy Spine Pst/Pstlat Appr 1 Vrt Sgm Lmbr |
| Spine | 22216 | Osteot Spi Pst/Pstlat Appr 1 Vrt Sgm Ea Vrt Sgm |
| Spine | 22220 | Osteotomy Spine W/Dsc Ant Appr 1 Vrt Sgm Crv |
| Spine | 22222 | Osteotomy Spine W/Dsc Ant Appr 1 Vrt Sgm Thrc |
| Spine | 22224 | Osteotomy Spine W/Dsc Ant Appr 1 Vrt Sgm Lumbar |
| Spine | 22325 | Optx&/Rdctj Vrt Fx&/Dislc Pst 1 Vrt Sgm Lm |
| Spine | 22326 | Optx&/Rdctj Vrt Fx&/Dislc Pst 1 Vrt Sgm Cr |
| Spine | 22327 | Optx&/Rdctj Vrt Fx&/Dislc Pst 1 Vrt Sgm Th |
| Spine | 22328 | Optx&/Rdctj Vrt Fx&/Dislc Pst 1 Vrt Sgm Ea |
| Spine | 22513 | Vertebral Compression Fracture Treatment (Kyphoplasty) |
| Spine | 22532 | Arthrodesis Lateral Extracavitary Thoracic |
| Spine | 22533 | Arthrodesis Lateral Extracavitary Lumbar |
| Spine | 22534 | Arthrodesis Lat Extracavitary Ea Addl Thrc/Lmbr |
| Spine | 22551 | Arthrd Ant Interbody Decompress Cervical Belw C2 |
| Spine | 22552 | Arthrd Ant Interdy Cervcl Belw C2 Ea Addl Ntrspc |
| Spine | 22554 | Arthrd Ant Interbody Min Dsc Crv Below C2 |
| Spine | 22556 | Arthrd Ant Interbody Min Dsc Thoracic |
| Spine | 22558 | Arthrd Ant Interbody Min Dsc Lumbar |
| Spine | 22585 | Arthrd Ant Ntrbd Min Dsc Ea Addl Interspace |
| Spine | 22590 | Arthrodesis Posterior Craniocervical |
| Spine | 22595 | Arthrodesis Posterior Atlas-Axis C1-C2 |
| Spine | 22600 | Arthrd Pst/Pstlat Tq 1Ntrspc Crv Belw C2 Segment |
| Spine | 22610 | Arthrodesis Posterior/Pstlat Tq 1Ntrspc Thoracic |
| Spine | 22612 | Arthrodesis Posterior/Pstlat Tq 1Ntrspc Lumbar |
| Spine | 22614 | Arthrodesis Pst/Pstlat Tq 1Ntrspc Ea Addl Ntrspc |
| Spine | 22630 | Arthrodesis Posterior Interbody 1 Ntrspc Lumbar |
| Spine | 22632 | Arthrodesis Posterior Interbody 1 Ntrspc Ea Addl |
| Spine | 22633 | Arthrodesis Combined Tq 1Ntrspc Lumbar |
| Spine | 22634 | Arthrodesis Cmbn Tq 1Ntrspc Each Additional |
| Spine | 22800 | Arthrodesis Posterior Spinal Dfrm <6 Vrt Sgm |
| Spine | 22802 | Arthrodesis Posterior Spinal Dfrm 7-12 Vrt Sgm |
| Spine | 22804 | Arthrodesis Posterior Spinal Dfrm 13+ Vrt Sgm |
| Spine | 22808 | Arthrodesis Anterior Spinal Dfrm 2-3 Vrt Sgm |
| Spine | 22810 | Arthrodesis Anterior Spinal Dfrm 4-7 Vrt Sgm |
| Spine | 22812 | Arthrodesis Anterior Spinal Dfrm 8+ Vrt Sgm |
| Spine | 22818 | Arthrodesis Procedures for Spine Deformity |
| Spine | 22819 | arthrodesis procedures for spine deformities, such as scoliosis or kyphosis, involving kyphectomy |
| Spine | 22830 | Exploration Spinal Fusion |
| Spine | 22836 | Ant Thoracic Vrt Body Tethering <7 Vrt Segments |
| Spine | 22838 | Revj Rplcmt/Rmvl Thoracic Vrt Body Tethering |
| Spine | 22840 | Posterior Non-Segmental Instrumentation |
| Spine | 22841 | Internal Spinal Fixation Wiring Spinous Process |
| Spine | 22842 | Posterior Segmental Instrumentation 3-6 Vrt Seg |
| Spine | 22843 | Posterior Segmental Instrumentation 7-12 Vrt Seg |
| Spine | 22844 | Posterior Segmental Instrumentation 13/> Vrt Se |
| Spine | 22845 | Anterior Instrumentation 2-3 Vertebral Segments |
| Spine | 22846 | Anterior Instrumentation 4-7 Vertebral Segments |
| Spine | 22847 | Anterior Instrumentation 8/> Vertebral Segments |
| Spine | 22848 | Pelvic Fixation Other Than Sacrum |
| Spine | 22849 | Reinsertion Spinal Fixation Device |
| Spine | 22850 | Removal Posterior Nonsegmental Instrumentation |
| Spine | 22851 | Application of intervertebral biomechanical device(s) (e.g., synthetic cage(s), methylmethacrylate) to vertebral defect or interspace |
| Spine | 22852 | Removal Posterior Segmental Instrumentation |
| Spine | 22853 | Insj Biomchn Dev Intervertebral Dsc Spc W/Arthrd |
| Spine | 22854 | Insj Biomchn Dev Vrt Corpectomy Defect W/Arthrd |
| Spine | 22855 | Removal Anterior Instrumentation |
| Spine | 22856 | Total Disc Arthrp Ant Single Interspace Cervical |
| Spine | 22857 | Total Disc Arthrp Ant Single Interspace Lumbar |
| Spine | 22858 | Total Disc Arthrp Ant 2Nd Level Cervical |
| Spine | 22859 | Insj Biomchn Dev Ntrvrt Disc Space W/O Arthrd |
| Spine | 22860 | Total Disc Arthrp Ant Second Interspace Lumbar |
| Spine | 22862 | revision including replacement of a total disc arthroplasty (artificial disc) using an anterior approach, for a single interspace, in the lumbar spine |
| Spine | 22864 | Rmvl Tot Disc Arthrp Ant 1 Interspace Cervical |
| Spine | 22865 | Removal of total disc arthroplasty (artificial disc), anterior approach, single interspace |
| Spine | 22869 | Insj Stablj Dev W/O Dcmprn Lumbar Single Level |
| Spine | 22899 | Unlisted Procedure Spine |
| Spine | 27080 | Coccygectomy Primary |
| Spine | 27279 | Arthrodesis Si Joint Percutaneous/Min Invasive |
| Spine | 27280 | Arthrodesis Si Jt Opn W/Obtaining B1 Grf Instrmj |
| Spine | 32905 | Thoracoplasty Schede Type/Extrapleural |
| Spine | 62264 | Prq Lysis Epidural Adhesions Mult Sessions 1 Day |
| Spine | 62267 | Prq Aspir Pulposus/Intervertebral Disc/Pvrt Tiss |
| Spine | 62268 | Percutaneous Aspiration Spinal Cord Cyst/Syrinx |
| Spine | 62287 | Dcmprn Px Perq Nucleus Pulposus 1/Mlt Lvl Lumbar |
| Spine | 62350 | Impltj Revj/Rpsg Ithcl/Edrl Cath Pmp W/O Lam |
| Spine | 62355 | Rmvl Previously Implted Ithcl/Edrl Cath |
| Spine | 62361 | Impltj/Rplcmt Fs Non-Prgrbl Pump |
| Spine | 62362 | Impltj/Rplcmt Ithcl/Edrl Drug Nfs Prgrbl Pump |
| Spine | 62365 | Rmvl Subq Rsvr/Pump Intrathecal/Epidural Infus |
| Spine | 62380 | Ndsc Dcmprn Spinal Cord 1 W/Lamot Ntrspc Lumbar |
| Spine | 63001 | Lam W/O Facetec Foramot/Dsc 1/2 Vrt Sgm Crv |
| Spine | 63003 | Laminectomy W/O Ffd 1/2 Vert Seg Thoracic |
| Spine | 63005 | Laminectomy W/O Ffd 1/2 Vert Seg Lumbar |
| Spine | 63011 | Laminectomy W/O Ffd 1/2 Vert Seg Sacral |
| Spine | 63012 | Laminectomy W/Rmvl Abnormal Facets Lumbar |
| Spine | 63015 | Laminectomy W/O Ffd > 2 Vert Seg Cervical |
| Spine | 63016 | Laminectomy W/O Ffd > 2 Vert Seg Thoracic |
| Spine | 63017 | Laminectomy W/O Ffd > 2 Vert Seg Lumbar |
| Spine | 63020 | Lamnotmy Incl W/Dcmprsn Nrv Root 1 Intrspc Cervc |
| Spine | 63030 | Lamnotmy Incl W/Dcmprsn Nrv Root 1 Intrspc Lumbr |
| Spine | 63035 | Lamnotmy W/Dcmprsn Nrv Each Addl Crvcl/Lmbr |
| Spine | 63040 | laminotomy (hemilaminectomy) with decompression of nerve roots, including partial facetectomy, foraminotomy, and/or excision of a herniated intervertebral disc, re-exploration, single interspace, cervical |
| Spine | 63042 | Lamot Prtl Ffd Exc Disc Reexpl 1 Ntrspc Lumbar |
| Spine | 63044 | Lamot W/Prtl Ffd Hrna8 Reexpl 1 Ntrspc Ea Lmbr |
| Spine | 63045 | Lam Facetectomy & Foramotomy 1 Vrt Sgm Cervical |
| Spine | 63046 | Lam Facetectomy & Foramotomy 1 Vrt Sgm Thoracic |
| Spine | 63047 | Lam Facetectomy & Foramotomy 1 Vrt Sgm Lumbar |
| Spine | 63048 | Lam Facetectomy&Foramot 1 Vrt Sgm Ea Addl Sgm |
| Spine | 63050 | Lamop Cervical W/Dcmprn Spi Cord 2/> Vert Seg |
| Spine | 63051 | Lamoplasty Cervical Dcmprn Cord 2/> Seg Rcnstj |
| Spine | 63052 | Lam Facetec/Foramot Drg Arthrd Lumbar 1 Vrt Sgm |
| Spine | 63053 | Lam Facetec/Foramot Drg Arthrd Lmbr Ea Addl Sgm |
| Spine | 63055 | Transpedicular Dcmprn Spinal Cord 1 Seg Thoracic |
| Spine | 63056 | Transpedicular Dcmprn Spinal Cord 1 Seg Lumbar |
| Spine | 63057 | Transpedicular Dcmprn 1 Seg Ea Thoracic/Lumbar |
| Spine | 63064 | Costovertebral Dcmprn Spinal Cord Thoracic 1 Seg |
| Spine | 63066 | Transpedicular or Costovertebral Approach for Posterolateral Extradural Exploration/Decompression Procedures on the Spine and Spinal Cord |
| Spine | 63075 | Discectomy Ant Dcmprn Cord Cervical 1 Ntrspc |
| Spine | 63076 | Discectomy Ant Dcmprn Cord Cervical Ea Ntrspc |
| Spine | 63077 | Discectomy Ant Dcmprn Cord Thoracic 1 Ntrspc |
| Spine | 63078 | Discectomy Ant Dcmprn Cord Thoracic Ea Ntrspc |
| Spine | 63079 | Surgical removal of a thoracic disc herniation |
| Spine | 63080 | Excision by Laminectomy of Lesion Other Than Herniated Disk Procedures |
| Spine | 63081 | Vertebral Corpectomy Ant Dcmprn Cervical 1 Seg |
| Spine | 63082 | Vertebral Corpectomy Dcmprn Cervical Ea Seg |
| Spine | 63083 | Excision by Laminectomy of Lesion Other Than Herniated Disk Procedures |
| Spine | 63084 | percutaneous vertebral augmentation (PVA) for a vertebral compression fracture |
| Spine | 63085 | Vertebral Corpectomy Dcmprn Cord Thoracic 1 Seg |
| Spine | 63086 | Vertebral corpectomy (vertebral body resection), partial or complete, |
| Spine | 63087 | Anterior or Anterolateral Approach for Extradural Exploration/Decompression Procedures on the Spine and Spinal Cord |
| Spine | 63090 | Vcrpec Transprtl/Rpr Dcmprn Thrc Lmbr/Sac 1 Seg |
| Spine | 63091 | Vcrpec Transprtl/Rpr Dcmprn Thrc Lmbr/Sac Ea Seg |
| Spine | 63101 | Verteb Corpect Lat Xtrcavitary Dcmprn Thrc 1 Seg |
| Spine | 63102 | Lateral Extracavitary Approach for Extradural Exploration/Decompression Procedures on the Spine and Spinal Cord |
| Spine | 63103 | Vcrpec Lat Xtrcavitary Dcmprn Thrc/Lmbr Ea Seg |
| Spine | 63200 | Incision Procedures on the Spine and Spinal Cord |
| Spine | 63252 | Excision by Laminectomy of Lesion Other Than Herniated Disk Procedures |
| Spine | 63265 | Lam Exc/Evac Ispi Les Oth/Thn Neo Xdrl Cervical |
| Spine | 63266 | Lam Exc/Evac Ispi Les Oth/Thn Neo Xdrl Thoracic |
| Spine | 63267 | Lam Exc/Evac Ispi Lesion Oth/Thn Neo Xdrl Lumbar |
| Spine | 63271 | Lam Exc Ispi Les Oth/Thn Neo Idrl Thoracic |
| Spine | 63272 | Lam Exc Ispi Les Oth/Thn Neo Idrl Lumbar |
| Spine | 63277 | Laminectomy Bx/Exc Ispi Neo Xdrl Lumbar |
| Spine | 63650 | Prq Impltj Nstim Electrode Array Epidural |
| Spine | 63655 | Lam Impltj Nstim Eltrds Plate/Paddle Edrl |
| Spine | 63661 | Rmvl Spinal Nstim Eltrd Prq Array Incl Fluor |
| Spine | 63662 | Rmvl Spinal Nstim Eltrd Plate/Paddle Incl Fluor |
| Spine | 63663 | Revj Incl Rplcmt Nstim Eltrd Prq Ra Incl Fluor |
| Spine | 63664 | Revj Incl Rplcmt Nstim Eltrd Plt/Pdle Incl Fluor |
| Spine | 63685 | Insj/Rplcmt Spinal Npg/Rcvr Pocket Crtj&Connj |
| Spine | 63688 | Revj/Rmvl Impl Spi Npg/Rcvr Dtch Connj Eltrd Ra |
| Spine | 64561 | Prq Impltj Neurostim Eltrd Sacral Nrve W/Imaging |
| Spine | 64633 | Dstr Nrolytc Agnt Parverteb Fct Sngl Crvcl/Thora |
| Spine | 64634 | Dstr Nrolytc Agnt Parverteb Fct Addl Crvcl/Thora |
| Spine | 64635 | Dstr Nrolytc Agnt Parverteb Fct Sngl Lmbr/Sacral |
| Spine | 64636 | Dstr Nrolytc Agnt Parverteb Fct Addl Lmbr/Sacral |
| Spine | 64772 | Transection/Avulsion Oth Spinal Nrv Xdrl |
| Spine | S2351 | Diskectomy, anterior, with decompression of spinal cord and/or nerve root(s), including osteophytectomy; lumbar, each additional interspace |
| Knee | 27303 | Inc Deep W/Opng Bone Cortex Femur/Knee |
| Knee | 27305 | Fasciotomy Iliotibial Open |
| Knee | 27310 | Arthrt Kne W/Expl Drg/Rmvl Fb |
| Knee | 27331 | Arthrt Kne W/Jt Expl Bx/Rmvl Loose/Fb |
| Knee | 27332 | Arthrt W/Exc Semilunar Crtlg Knee Medial/Lat |
| Knee | 27333 | Arthrt W/Exc Semilunar Crtlg Knee Medial&Lat |
| Knee | 27334 | Arthrotomy W/Synovectomy Knee Anterior/Posterior |
| Knee | 27335 | Arthrt W/Synvct Kne Ant&Post W/Pop Area |
| Knee | 27340 | Excision Prepatellar Bursa |
| Knee | 27345 | Excision Synovial Cyst Popliteal Space |
| Knee | 27347 | Excision Lesion Meniscus/Capsule Knee |
| Knee | 27350 | Patellectomy/Hemipatellectomy |
| Knee | 27360 | Prtl Exc Bone Femur Prox Tibia&/Fibula |
| Knee | 27372 | Removal Foreign Body Deep Thigh/Knee |
| Knee | 27380 | Suture Infrapatellar Tendon Primary |
| Knee | 27381 | Sutr Infrapatellar Tdn 2 Rcnstj W/Fscal/Tdn Grf |
| Knee | 27403 | Arthrotomy W/Meniscus Repair Knee |
| Knee | 27405 | Rpr Primary Torn Ligm&/Capsule Knee Collateral |
| Knee | 27407 | Repair Primary Torn Ligm&/Capsule Knee Cruciat |
| Knee | 27415 | Osteochondral Allograft Knee Open |
| Knee | 27416 | Osteochondral Autograft Knee Open Mosaicplasty |
| Knee | 27418 | Anterior Tibial Tubercleplasty |
| Knee | 27420 | Rcnstj Dislocating Patella |
| Knee | 27422 | Rcnstj Dislc Patella W/Xtnsr Relignmt&/Musc Rl |
| Knee | 27425 | Lateral Retinacular Release Open |
| Knee | 27427 | Ligamentous Reconstruction Knee Extra-Articular |
| Knee | 27428 | Ligamentous Reconstruction Knee Intra-Articular |
| Knee | 27429 | Ligmous Rcnstj Agmntj Kne Intra-Articular Xtr |
| Knee | 27435 | Capsulotomy Posterior Capsular Release Knee |
| Knee | 27437 | Arthroplasty Patella W/O Prosthesis |
| Knee | 27438 | Arthroplasty Patella W/Prosthesis |
| Knee | 27442 | Arthroplasty Fem Condyles/Tibial Plateau Knee |
| Knee | 27446 | Arthrp Knee Condyle&Plateau Medial/Lat Cmprt |
| Knee | 27447 | Arthrp Kne Condyle&Platu Medial&Lat Compartments |
| Knee | 27455 | Osteot Prox Tibia Fib Exc/Osteot Before Epiphysl |
| Knee | 27457 | Osteot Prox Tibia Fib Exc/Osteot After Epiphysl |
| Knee | 27470 | Rpr Non/Mal Femur Dstl H/N W/O Grf |
| Knee | 27475 | Arrest Epiphyseal Distal Femur |
| Knee | 27477 | Arrest Epiphyseal Tibia & Fibula Proximal |
| Knee | 27479 | Arrst Epiphysl Cmbn Dstl Femur Prox Tibfib |
| Knee | 27486 | Revj Total Knee Arthrp W/Wo Algrft 1 Component |
| Knee | 27487 | Revj Tot Knee Arthrp Fem&Entire Tibial Compone |
| Knee | 27498 | Dcmprn Fasciotomy Thigh&/Knee Mlt Compartments |
| Knee | 27509 | Prq Skeletal Fixj Femoral Fx Distal End |
| Knee | 27514 | Open Tx Femoral Fracture Distal Med/Lat Condyle |
| Knee | 27524 | Optx Patllr Fx W/Int Fixj/Patllc&Soft Tiss Rpr |
| Knee | 27535 | Open Tx Tibial Fracture Proximal Unicondylar |
| Knee | 27536 | Optx Tibial Fx Prox Bicondylar W/Wo Int Fixj |
| Knee | 27540 | Open Tx Intercondylar Spine/Tubrst Fracture Knee |
| Knee | 27556 | Open Tx Knee Dislocation W/O Ligamentous Repair |
| Knee | 27557 | Open Tx Knee Dislocation W/Ligamentous Repair |
| Knee | 27558 | Open Tx Knee Dislocation W/Repair/Reconstruction |
| Knee | 27566 | Optx Patellar Dislc W/Wo Prtl/Tot Patellectomy |
| Knee | 27570 | Manipulation Knee Joint Under General Anesthesia |
| Knee | 27580 | Arthrodesis Knee Any Technique |
| Knee | 27784 | Open Treatment Proximal Fibula/Shaft Fracture |
| Knee | 27832 | Open Tx Prox Tibfib Joint Dislocate Exc Prox Fib |
| Knee | 29850 | Arthroscopy Aid Tx Spine&/Fx Knee W/O Fixj |
| Knee | 29851 | Arthroscopy Aid Tx Spine&/Fx Knee W/Fixj |
| Knee | 29855 | Arthrs Aid Tibial Fracture Proximal Unicondylar |
| Knee | 29856 | Arthrs Aid Tibial Fx Prox Unicondylar Bicondylar |
| Knee | 29866 | Arthroscopy Knee Osteochondral Agrft Mosaicplast |
| Knee | 29867 | Arthroscopy Knee Osteochondral Allograft |
| Knee | 29868 | Arthroscopy Knee Meniscal Trnsplj Med/Lat |
| Knee | 29870 | Arthroscopy Knee Diagnostic W/Wo Synovial Bx Spx |
| Knee | 29871 | Arthroscopy Knee Infection Lavage & Drainage |
| Knee | 29873 | Arthroscopy Knee Lateral Release |
| Knee | 29874 | Arthroscopy Knee Removal Loose/Foreign Body |
| Knee | 29875 | Arthroscopy Knee Synovectomy Limited Spx |
| Knee | 29876 | Arthroscopy Knee Synovectomy 2/>Compartments |
| Knee | 29877 | Arthrs Knee Debridement/Shaving Artclr Crtlg |
| Knee | 29879 | Arthrs Knee Abrasion Arthrp/Mlt Drlg/Microfx |
| Knee | 29880 | Arthrs Knee W/Meniscectomy Med&Lat W/Shaving |
| Knee | 29881 | Arthrs Kne Surg W/Meniscectomy Med/Lat W/Shvg |
| Knee | 29882 | Arthroscopy Knee W/Meniscus Rpr Medial/Lateral |
| Knee | 29883 | Arthroscopy Knee W/Meniscus Rpr Medial&Lateral |
| Knee | 29884 | Arthroscopy Knee W/Lysis Adhesions W/Wo Manj Spx |
| Knee | 29885 | Arthrs Knee Drill Osteochondritis Dissecans Grfg |
| Knee | 29886 | Arthrs Knee Drilling Osteochond Dissecans Lesion |
| Knee | 29887 | Arthrs Knee Drlg Osteochond Dissecans Int Fixj |
| Knee | 29888 | Arthrs Aided Ant Cruciate Ligm Rpr/Agmntj/Rcnstj |
| Knee | 29889 | Arthrs Aided Pst Cruciate Ligm Rpr/Agmntj/Rcnstj |
| Knee | S2112 | Knee arthroscp harv |
| Hip | 20922 | Fascia Lata Graft Incision & Area Exposure |
| Hip | 27000 | Tenotomy Adductor Hip Percutaneous Spx |
| Hip | 27001 | Tenotomy Adductor Hip Open |
| Hip | 27005 | Tenotomy Hip Flexor Open Separate Procedure |
| Hip | 27006 | Tenotomy Abductors&/Extensor Hip Open Spx |
| Hip | 27025 | Fasciotomy Hip/Thigh Any Type |
| Hip | 27027 | Decompression Fasciotomy Pelvic Compartment Uni |
| Hip | 27030 | Arthrotomy Hip W/Drainage |
| Hip | 27033 | Arthrotomy Hip Exploration/Removal Foreign Body |
| Hip | 27036 | Capslctomy/Capsul Hip W/Rls Hip Flxr Musc |
| Hip | 27048 | Exc Tumor Soft Tissue Pelvis & Hip Subfasc <5Cm |
| Hip | 27054 | Arthrotomy W/Synovectomy Hip Joint |
| Hip | 27060 | Excision Ischial Bursa |
| Hip | 27062 | Excision Trochanteric Bursa/Calcification |
| Hip | 27066 | Excision Bone Cyst/Benign Tumor Deep |
| Hip | 27100 | Tr Xtrnl Oblq Musc Trchntr W/Fscal/Tdn Xtn Grf |
| Hip | 27110 | Transfer Iliopsoas Greater Trochanter Femur |
| Hip | 27125 | Hemiarthroplasty Hip Partial |
| Hip | 27130 | Arthrp Acetblr/Prox Fem Prostc Agrft/Algrft |
| Hip | 27132 | Conv Prev Hip Tot Hip Arthrp W/Wo Agrft/Algrft |
| Hip | 27134 | Revj Tot Hip Arthrp Bth W/Wo Agrft/Algrft |
| Hip | 27137 | Revj Tot Hip Arthrp Actblr W/Wo Agrft/Algrft |
| Hip | 27138 | Revj Tot Hip Arthrp Fem Only W/Wo Algrft |
| Hip | 27140 | Osteotomy&Transfer Greater Trochanter Spx |
| Hip | 27146 | Osteotomy Iliac Acetabular/Innominate Bone |
| Hip | 27147 | Osteotomy Iliac Acetabular/Innominate Hip Rdctj |
| Hip | 27151 | Osteotomy Iliac Acetabular/Innominate Fem Osteot |
| Hip | 27165 | Osteot Intertrchntric/Subtrchntric W/Int/Xtrnl |
| Hip | 27176 | Tx Slp Fem Epiphysis Single/Multipl Pinning Situ |
| Hip | 27179 | Optx Slp Fem Epiphysis Ostpl Fem Nck Heyman Px |
| Hip | 27187 | Proph Tx N/P/Pltwr W/Wo Mma Fem Nck & Prox Femur |
| Hip | 27235 | Prq Skel Fixj Femoral Fx Prox End Neck |
| Hip | 27236 | Optx Fem Fx Prox End Nck Int Fixj/Prostc Rplcmt |
| Hip | 27245 | Tx Inter/Pr/Subtrchntric Fem Fx Imed Impltscrew |
| Hip | 27248 | Open Treatment Greater Trochanteric Fracture |
| Hip | 27257 | Tx Spon Hip Dislc Abdct Splnt/Trcj W/Manj Anes |
| Hip | 27269 | Open Tx Femoral Fracture Proximal End Head |
| Hip | 27275 | Manipulation Hip Joint General Anesthesia |
| Hip | 27391 | Tenotomy Opn Hamstring Knee Hip Multiple 1 Leg |
| Hip | 27454 | Osteot Mlt W/Relignmt Imed Rod Fem Shft |
| Hip | 29860 | Arthroscopy Hip Diagnostic W/Wo Synovial Byp Spx |
| Hip | 29861 | Arthroscopy Hip Surgical W/Removal Loose/Fb |
| Hip | 29862 | Arthrs Hip Debridement/Shaving Articular Crtlg |
| Hip | 29863 | Arthroscopy Hip Surgical W/Synovectomy |
| Hip | 29914 | Arthroscopy Hip W/Femoroplasty |
| Hip | 29915 | Arthroscopy Hip W/Acetabuloplasty |
| Hip | 29916 | Arthroscopy Hip W/Labral Repair |
| Hip | 64712 | Neurp Major Prph Nrv Opn Arm/Leg Sciatic Nrv |
| Hip | 64840 | Suture Posterior Tibial Nerve |
| Hip | 64858 | Suture Sciatic Nerve |
| Hip | S2115 | Periacetabular osteotomy |
| Hip | S2118 | Total Hip resurfacing |
| Hip | S2325 | Hip core decompression |
| Shoulder | 23000 | Removal Subdeltoid Calcareous Deposits Open |
| Shoulder | 23040 | Arthrotomy Glenohumeral Jt Expl/Drg/Rmvl Fb |
| Shoulder | 23044 | Arthrt Acromclav Strnclav Jt Expl/Drg/Rmvl Fb |
| Shoulder | 23101 | Arthrt Acromclav/Strnclav Jt W/Bx&/Exc Crtlg |
| Shoulder | 23120 | Claviculectomy Partial |
| Shoulder | 23125 | Claviculectomy Total |
| Shoulder | 23130 | Acromioplasty/Acromionectomy Prtl +-Ligament Rls |
| Shoulder | 23140 | Exc/Curtg Bone Cyst/Benign Tumor Clav/Scapula |
| Shoulder | 23170 | Sequestrectomy Clavicle |
| Shoulder | 23180 | Partial Excision Bone Clavicle |
| Shoulder | 23184 | Partial Excision Bone Proximal Humerus |
| Shoulder | 23190 | Ostectomy Scapula Partial |
| Shoulder | 23195 | Resection Humeral Head |
| Shoulder | 23395 | Muscle Transfer Shoulder/Upper Arm Single |
| Shoulder | 23397 | Muscle Transfer Shoulder/Upper Arm Multiple |
| Shoulder | 23400 | Scapulopexy |
| Shoulder | 23405 | Tenotomy Shoulder Area 1 Tendon |
| Shoulder | 23406 | Tenotomy Shoulder Multiple Thru Same Incision |
| Shoulder | 23410 | Open Repair Of Rotator Cuff Acute |
| Shoulder | 23412 | Open Repair Of Rotator Cuff Chronic |
| Shoulder | 23415 | Coracoacromial Ligament Releas W/Woacromioplasty |
| Shoulder | 23420 | Reconstruction Rotator Cuff Avulsion Chronic |
| Shoulder | 23430 | Tenodesis Long Tendon Biceps |
| Shoulder | 23440 | Resection/Transplantation Long Tendon Biceps |
| Shoulder | 23450 | Capsulorrhaphy Anterior Putti-Platt/Magnuson |
| Shoulder | 23455 | Capsulorrhaphy Anterior W/Labral Repair |
| Shoulder | 23460 | Capsulorrhaphy Anterior With Bone Block |
| Shoulder | 23462 | Capsulorrhaphy Anterior W/Coracoid Process Tr |
| Shoulder | 23465 | Capsulorrhaphy Glenohumeral Jt Pst W/Wo Bone Blk |
| Shoulder | 23466 | Capsulorrhaphy Glenohumrl Jt Multi-Dirional Ins |
| Shoulder | 23470 | Arthroplasty Glenohumrl Jt Hemiarthroplasty |
| Shoulder | 23472 | Arthroplasty Glenohumeral Joint Total Shoulder |
| Shoulder | 23473 | Revis Shoulder Arthrplsty Humeral/Glenoid Compnt |
| Shoulder | 23474 | Revis Shoulder Arthrplsty Humeral&Glenoid Compnt |
| Shoulder | 23515 | Open Tx Clavicular Fracture Internal Fixation |
| Shoulder | 23532 | Optx Strnclav Dislc Acute/Chronic W/Fascial Grf |
| Shoulder | 23550 | Open Tx Acromioclavicular Dislc Acute/Chronic |
| Shoulder | 23552 | Optx Acromclav Dislc Acute/Chronic W/Fascial Grf |
| Shoulder | 23585 | Open Tx Scapular Fx W/Int Fixation When Pfrmd |
| Shoulder | 23615 | Optx Prox Humeral Fx W/Int Fixj Rpr Tuberosity |
| Shoulder | 23630 | Optx Greater Humeral Tuberosity Fx W/Int Fixj |
| Shoulder | 23700 | Mnpj W/Anes Shoulder Jt Appl Fixation Apparatus |
| Shoulder | 24073 | Exc Tumor Soft Tiss Upper Arm/Elbw Subfasc 5Cm/> |
| Shoulder | 24140 | Partial Excision Bone Humerus |
| Shoulder | 24305 | Tendon Lengthening Upper Arm/Elbow Ea Tendon |
| Shoulder | 24310 | Tenotomy Open Elbow To Shoulder Each Tendon |
| Shoulder | 24332 | Tenolysis Triceps |
| Shoulder | 24400 | Osteotomy Humerus W/Wo Internal Fixation |
| Shoulder | 24420 | Osteoplasty Humerus |
| Shoulder | 24515 | Optx Humeral Shft Fx W/Plate/Screws W/Wocerclage |
| Shoulder | 29805 | Diagnostic Arthroscopy Shoulder +- Synovial Bx |
| Shoulder | 29806 | Surgical Arthroscopy Shoulder Capsulorrhaphy |
| Shoulder | 29807 | Surgical Arthroscopy Shoulder Repair Slap Lesion |
| Shoulder | 29819 | Surgical Arthroscopy Shoulder Removal Loose/Fb |
| Shoulder | 29820 | Surgical Arthroscopy Shoulder Prtl Synovectomy |
| Shoulder | 29821 | Surgical Arthroscopy Shoulder Compl Synovectomy |
| Shoulder | 29822 | Surgical Arthroscopy Shoulder Lmtd Dbrdmt 1/2 |
| Shoulder | 29823 | Surgical Arthroscopy Shoulder Xtnsv Dbrdmt 3+ |
| Shoulder | 29824 | Surgical Arthroscopy Shoulder Dstl Claviculc |
| Shoulder | 29825 | Surgical Arthroscopy Shoulder W/Lss&Rescj Ads |
| Shoulder | 29826 | Surgical Arthroscopy Sho W/Coracoacrm Ligm Rls |
| Shoulder | 29827 | Surgical Arthroscopy Shoulder W/Rotator Cuff Rpr |
| Shoulder | 29828 | Surgical Arthroscopy Shoulder Biceps Tenodesis |
| Shoulder | 64713 | Neurp Major Prph Nrv Opn Arm/Leg Brach Plexus |
| Shoulder | 7701 | Sequestrectomy, scapula, clavicle, and thorax |
| Shoulder | 95928 | Ctr Motor Ep Std Transcrnl Motor Stimj Upr Limbs |
| Shoulder | C9781 | Arthroscopy Shoulder surg; w/imp sa spacer |
| Shoulder | L6641 | Excursion amplifier pulley t |

# Table S6. Coding algorithms used to identify surgical procedures classified as low-value care, with supporting evidence.

| **Indicator** | **Evidence** | **Inclusion criteria** | | **Exclusion criteria** | | **Prerequisite^a^** |
| --- | --- | --- | --- | --- | --- | --- |
|  |  | **Diagnoses**  **(ICD-10 codes)** | **Procedure codes**  **(HCPCS/CPT/**  **ICD-10-PCS)** | **Diagnoses**  **(ICD-10 codes)** | **Procedure codes**  **(HCPCS/CPT/**  **ICD-10-PCS)** |  |
| Spinal surgery for spinal conditions | High-quality evidence against spinal fusion [4]  No supporting evidence for disc replacement [5]  Evidence supporting non-surgical care compared to surgery [6] | M48.00, M48.061, M48.062, M48.07, M48.08, M51.06, M51.16, M51.17, M51.26, M51.27, M51.36, M51.37, M47.26, M47.27, M43.8, M43.9, M43.5X5, M43.5X6, M43.5X7, M43.5X8, M43.5X9, M47.20, M47.28, M47.816, M47.817, M47.818, M47.819, M47.896, M47.897, M47.898, M47.899, M47.9, M51.34, M51.35, M51.86, M51.87, M53.86, M53.87, M53.88, M54.30, M54.31, M54.32, M54.40, M54.41, M54.42, M54.5, M54.89, M54.9, M99.03, M99.04, M99.23, M99.33, M99.43, M99.53, M99.63, M99.73, M99.83, M99.84, S33.5XXA, S33.5XXD, S33.5XXS, S33.6XXA, S33.6XXD, S33.6XXS, S33.8XXA, S33.8XXD, S33.8XXS, S33.9XXA, S33.9XXD, S33.9XXS | 22224, 22533, 22558, 22586, 22612, 22614, 22630, 22632, 22633, 22800, 22802, 22804, 22808, 22810, 22857, 22862, 22867, 63005, 63011, 63012, 63017, 63047, 63048, 63056, 63087, 63088, 63090, 63091, 63102, 63103, 63170, 62287, 63030, 63035, 63042, 22534, 0275T, 63267, 63272, 63173, 63185, 63190, 63191, 62380, 63001, 63003, 63015, 63016, 63020, 63040, 63043, 63044, 63045, 63046, 63052, 63053, 63055, 63057, 63064, 63075, 63076, 63077, 63078, 63081, 63082, 63085, 63086, 22595, 22600, 22610, 22551, 22552, 22554, 22556, 22585, 22634, 22856, 22858, 22861, 22864, 20930, 20931, 20936, 20937, 20938, 20939, 0221T, 22840, 22841, 22842, 22843, 22844, 22845, 22846, 22847, 22853, 22854, 22859, 22859 | G83.4, R15, S32.0, N31, C41.2, M48.56, M84.58, R32, M48.46, M84.48, M84.40, M84.60, M48.57, M87, S22.08, C00-C43, C45-C96, M46.2-M46.3, S34 | NA | NA |
| Meniscectomy for knee degenerative joint disease | Moderate- to high-quality evidence for lack of benefit over sham treatments [7-9]  Evidence supporting non-surgical care compared to surgery [7, 9, 10] | M17 | 29881, 27403, 29868, 29880, 29882, 29883  0SBC4ZZ, 0SBD4ZZ | NA | 0SRC06A, 0SRC06Z, 0SRC07Z, 0SRC0J9, 0SRD06Z, 0SRD07Z, 0SRD0J9 | NA |
| Knee arthroplasty for knee osteoarthritis | Evidence supporting non-surgical care [11, 12] | M17.0, M17.10, M17.11, M17.12, M17.2, M17.30, M17.31, M17.32, M17.4, M17.5, M17.9 | 27438, 27440, 27441, 27442, 27443, 27445, 27446, 27447 | NA | M80, M87, C79.51, C41.9 | No physical therapy within 6 months before surgery |
| Hip arthroplasty for hip osteoarthritis | Evidence supporting non-surgical care [11] | M16.0, M16.10, M16.11, M16.12, M16.2, M16.30, M16.31, M16.32, M16.4, M16.50, M16.51, M16.52, M16.6, M16.7, M16.9 | 27125, 27130, 27132 | NA | M84.459, M84.559, S72.0, S32.4, M87, M80, M84.359, M84.58, S72.1, S72.2, C79.51, M84.30, M84.40, M84.48, M84.50, C41.4, C41.9 | No physical therapy within 6 months before surgery |
| Rotator cuff repair for partial tears | No evidence supporting superiority of rotator cuff repair [13]  Evidence supporting non-surgical care [14] | S46.01, M75.11, M75.10 | 23410, 23412, 23420, 29827 | NA | M75.12 | No physical therapy within 6 months before surgery |
| Replacement for shoulder osteoarthritis | No evidence supporting superiority of shoulder replacement [15]  Evidence supporting non-surgical care [16] | M19.0, M19.11, M19.219, M19.90, M13.119 | 23472, 23474, 23470 | NA | NA | No physical therapy within 6 months before surgery |
| Arthroscopic release or manipulation under anesthesia for frozen shoulder | Evidence supporting non-surgical care [17] | M75.0, M25.51 | 23700, 29807, 29825 | NA | NA | No physical therapy within 6 months before surgery |
| Shoulder arthroscopy / distal clavicle resection for rotator cuff disorders or arthritis | High-quality evidence for lack of benefit over placebo surgery [18, 19]  Evidence supporting non-surgical care compared to surgery [18-20] | M19.02, M25.51, M75.4, M75.5, M75.11, M25.51, M75.3 | 23120, 29824, 29826, 29822, 29823, 23130,  0RRJ00Z, 0RRK00Z, 0RRJ0JZ, 0RRK0JZ, 0RRJ07Z, 0RRK07Z, 0RRJ0KZ, 0RRK0KZ, 0RRJ0J6, 0RRK0J6, 0RRJ0J7, 0RRK0J7 | NA | NA | No physical therapy within 6 months before surgery |
| Acromioplasty / acromiotomy for rotator cuff disorders | High-quality evidence for lack of benefit over placebo surgery [19]  No significant difference between surgery with versus without acromioplasty [21] | M25.51, M75.4, M75.5, M75.11, M25.51, M75.3 | 23130 | NA | NA | NA |
| Shoulder labral repair for labral tears | Evidence supporting non-surgical care [22]  No evidence regarding the effectiveness of surgical management for post-traumatic chronic shoulder instability [22] | S43.43, M75.8, S43.0 | 29807, 29828, 29806 | NA | NA | No physical therapy within 6 months before surgery |

**Abbreviations:** CPT, Current Procedural Terminology; HCPCS, Healthcare Common Procedure Coding System; ICD-10-PCS, International Classification of Diseases, 10th Revision, Procedure Coding System. **Note:** a. Surgeries were classified as low-value care only if no physical therapy sessions were recorded in the 6 months immediately preceding the procedure. For patients in the intervention group, this assessment included both digital and in-person therapy sessions (CPT codes: 97110, 97112, 97113, 97124, 97140), whereas for the comparator group it was based solely on claims for in-person physical therapy (same CPT codes).

# Table S7. Clinical outcome measures.

| **Clinical outcome** | **Measure** |
| --- | --- |
| **Pain** | Numerical Pain Rating Scale (NPRS) through the question: “Please rate your average pain over the last 7 days” (range 0-10; higher scores denote greater pain) [23] |
| **Work productivity and Daily Activities** | Work Productivity and Activity Impairment questionnaire - General Health v2.0 evaluated overall work productivity (WPAI-overall) and daily activities impairment (WPAI-activities) (range 0-100; higher scores denote greater impairment) [24] |
| **Satisfaction** | Through the question: “On a scale from 0 to 10, how likely is it that you would recommend this intervention to a friend or neighbor?”. |

# Table S8. Total counts and counts per 1,000 individuals of surgeries and low-value surgeries: overall and stratified by pain sites.

| **Therapy Area** | **Outcome Type** | **IG** | | **CG** | | **Difference** | | |
| --- | --- | --- | --- | --- | --- | --- | --- | --- |
|  |  | **Count** | **Counts per 1,000 individuals** | **Count** | **Counts per 1,000 individuals** | **%** | **Count** | **Counts per 1,000 individuals** |
| **Overall** | **Any surgery** | 123 | 58.7 | 328 | 156.6 | 62.5 | 205 | 97.9 |
|  | **Low-value surgery** | 13 | 6.2 | 81 | 38.7 | 84.0 | 68 | 32.5 |
| **Spine** | **Any surgery** | 42 | 20.0 | 88 | 42.0 | 52.3 | 46 | 22.0 |
|  | **Low-value surgery** | 9 | 4.2 | 42 | 19.8 | 78.6 | 33 | 15.6 |
| **Knee** | **Any surgery** | 43 | 20.5 | 154 | 73.5 | 72.1 | 111 | 53.0 |
|  | **Low-value surgery** | 2 | 1.0 | 25 | 11.9 | 92.0 | 23 | 11.0 |
| **Hip** | **Any surgery** | 19 | 9.1 | 46 | 22.0 | 58.7 | 27 | 12.9 |
|  | **Low-value surgery** | 2 | 0.9 | 10 | 4.8 | 80.0 | 8 | 3.8 |
| **Shoulder** | **Any surgery** | 19 | 9.1 | 40 | 19.1 | 52.5 | 21 | 10.0 |
|  | **Low-value surgery** | 0 | 0 | 4 | 1.9 | 100.0 | 4 | 1.9 |

**Abbreviations:** CG, comparator group; IG, intervention group

# Table S9. Sensitivity analysis results using augmented inverse propensity weighting (AIPW).

|  |  | **N** | **Estimate (**95%CI) | **Standard Error** |
| --- | --- | --- | --- | --- |
| **Overall surgery** | **Risk on IG** | 3,443 | 0.066 (0.051; 0.081) | 0.008 |
|  | **Risk on CG** | 15,171 | 0.146 (0.141; 0.151) | 0.003 |
|  | **Risk difference** | 18,614 | -0.080 (-0.096; -0.064) | 0.008 |
|  | **Risk Ratio** | 18,614 | 0.451 (0.354; 0.570) | 0.120 |
| **Low-value surgery** | **Risk on IG** | 3,443 | 0.006 (0.002; 0.034) | 0.002 |
|  | **Risk on CG** | 15,171 | 0.031 (0.028; 0.034) | 0.001 |
|  | **Risk difference** | 18,614 | -0.025 (-0.030; -0.021) | 0.002 |
|  | **Risk Ratio** | 18,614 | 0.187 (0.100; 0.350) | 0.319 |

**Abbreviations:** CG, comparator group; IG, intervention group.

**Note:** N reflects the number of possible index events considered when creating the matched population.

Sensitivity analyses were performed using the Augmented Inverse Propensity Weighting (AIPW) estimator, a doubly robust method that combines propensity score weighting with outcome regression modeling. Propensity scores were estimated from baseline covariates to adjust for confounding, and outcome models predicted the probability of surgery. This combination provides unbiased and efficient effect estimates if either model is correctly specified.
The table reports adjusted probabilities (risks) of surgery in the IG and CG, the absolute risk difference, and the relative risk (risk ratio), each with corresponding 95% CIs and standard errors. The analyses were performed separately for overall surgery and low-value surgery. Estimates represent marginal effects, adjusted for the specified covariates through both the propensity weighting and outcome regression components of the AIPW framework.

# Table S10. Negative control analysis comparing numbers of non-MSK-related elective procedures (cataract surgery and colonoscopy) between the intervention and comparator groups.

| **Procedure** | **Pre-index** | | | **Post-index** | | |
| --- | --- | --- | --- | --- | --- | --- |
|  | **IG, mean (n/N)** | **CG, mean (n/N)** | ***P*-value** | **IG,**  **mean (n/N)** | **CG,**  **mean (n/N)** | ***P*-value** |
| **Cataract surgery^1^** | 0.01 (27/2,095) | 0.01 (28/2,095) | 0.913 | 0.01 (28/2,095) | 0.01 (30/2,095) | 1.00 |
| **Colonoscopy^2^** | 0.07 (147/2,095) | 0.07 (151/2,095) | 0.525 | 0.06 (126/2,095) | 0.05 (104/2,095) | 0.159 |

**Notes: ^1^**CPT codes included: ‘66982’, ‘66983’, ‘66984’. **^2^**CPT codes: ‘45378’, ‘45380’.

**Abbreviations:** CG, comparator group; CPT, Current Procedural Terminology; IG, intervention group.

# Table S11. Total counts of low-value surgeries, overall and by pain site, across alternative preoperative observation windows (3, 6, 9, and 12 months) using different low-value care criteria. Values represent the number of low-value surgeries identified in the intervention group (IG) and comparison group (CG), along with the absolute and percentage differences between groups.

|  | **3 months** | | | **6 months** | | | **9 months** | | | **12 months** | | |
| --- | --- | --- | --- | --- | --- | --- | --- | --- | --- | --- | --- | --- |
| **Pain site** | **IG**  **counts** | **CG**  **counts** | **Difference**  **counts (%)** | **IG**  **counts** | **CG**  **counts** | **Difference**  **counts (%)** | **IG**  **counts** | **CG**  **counts** | **Difference**  **counts (%)** | **IG**  **counts** | **CG**  **counts** | **Difference**  **counts (%)** |
| **Overall** | 20 | 87 | 67 (77.0) | 13 | 81 | 68 (84.0) | 11 | 75 | 64 (85.3) | 9 | 71 | 62 (87.3) |
| **Spine** | 9 | 42 | 33 (78.6) | 9 | 42 | 33 (78.6) | 9 | 42 | 33 (78.6) | 9 | 42 | 33 (78.6) |
| **Knee** | 6 | 27 | 21 (77.8) | 2 | 25 | 23 (92.0) | 1 | 22 | 21 (95.5) | 0 | 19 | 19 (100.0) |
| **Hip** | 2 | 13 | 11 (84.6) | 2 | 10 | 8 (80.0) | 1 | 10 | 9 (90.0) | 0 | 9 | 9 (100.0) |
| **Shoulder** | 3 | 5 | 2 (40.0) | 0 | 4 | 4 (100.0) | 0 | 1 | 1 (100.0) | 0 | 1 | 1 (100.0) |

**Abbreviations:** CG, comparator group; IG, intervention group.

**Note:** The preoperative observation window was applied to surgical procedures for which conservative therapy is clinically indicated, including: knee arthroplasty for knee osteoarthritis; hip arthroplasty for hip osteoarthritis; rotator cuff repair for partial tears; shoulder replacement for osteoarthritis; arthroscopic release or manipulation under anesthesia for frozen shoulder; shoulder arthroscopy or distal clavicle resection for rotator cuff disorders or arthritis; and shoulder labral repair for labral tears. Surgeries were classified as low-value care only when no conservative therapy sessions were recorded during the 3-, 6-, 9-, or 12-month period immediately preceding the procedure. For patients in the intervention group, this assessment included both digital and in-person therapy sessions (CPT codes: 97110, 97112, 97113, 97124, 97140), whereas for the comparator group, it was based exclusively on claims for in-person conservative therapies (same CPT codes).

# Table S12. Surgery and low-value surgery stratified by social deprivation level and age groups.

|  | **Outcome** | **IG, % (n/N)** | **CG, % (n/N)** | **Relative difference, %** | **RR (95% CI)** |
| --- | --- | --- | --- | --- | --- |
| **Social Deprivation Index (SDI)** | | | | | |
| **SD1 C1 (0-25)**  **(least deprived)** | **Any surgery** | 6.4 (53/822) | 12.9 (107/851) | 49.2 | 0.51 (0.37;0.70) |
|  | **Low-value surgery** | 0.9 (7/822) | 3.4 (29/851) | 75.0 | 0.25 (0.11;0.57)) |
| **SDI C2 (26-50)** | **Any surgery** | 4.2 (24/567) | 14.0 (80/570) | 70.0 | 0.30 (0.19;0.47) |
|  | **Low-value surgery** | 0.5 (3/567) | 3.5 (20/570) | 84.9 | 0.15 (0.05;0.50) |
| **SDI C3 (51-75)** | **Any surgery** | 4.6 (19/414) | 13.3 (58/436) | 65.4 | 0.34 (0.21;0.57) |
|  | **Low-value surgery** | 0.5 (2/414) | 4.1 (18/436) | 88.3 | 0.12 (0.03;0.50) |
| **SDI C4 (76-100)**  **(most deprived)** | **Any surgery** | 6.5 (19/292) | 13.0 (31/238) | 50.0 | 0.50 (0.29;0.86) |
|  | **Low-value surgery** | 0.3 (1/292) | 2.9 (7/238) | 88.4 | 0.12 (0.01;0.94) |
| **Age group (years)** | | | | | |
| **18-39** | **Any surgery** | 3.0 (8/265) | 7.2 (19/265) | 58.3 | 0.42 (0.19;0.94) |
|  | **Low-value surgery** | 0.0 (0/265) | 3.0 (8/265) | 100.0 | - |
| **40-59** | **Any surgery** | 4.8 (60/1,251) | 11.5 (144/1,251) | 58.3 | 0.42 (0.31;0.56) |
|  | **Low-value surgery** | 0.4 (5/1,251) | 3.1 (39/1,251) | 87.2 | 0.13 (0.05;0.32) |
| **≥60** | **Any surgery** | 8.1 (47/579) | 19.5 (113/579) | 58.5 | 0.42 (0.30;0.57) |
|  | **Low-value surgery** | 1.4 (8/579) | 4.7 (27/579) | 70.4 | 0.30 (0.14;0.65) |

**Abbreviations:** CG, Comparator group; CI, Confidence interval; IG, Intervention group; RR, Risk ratio; SDI, social deprivation index.

(A) (B)
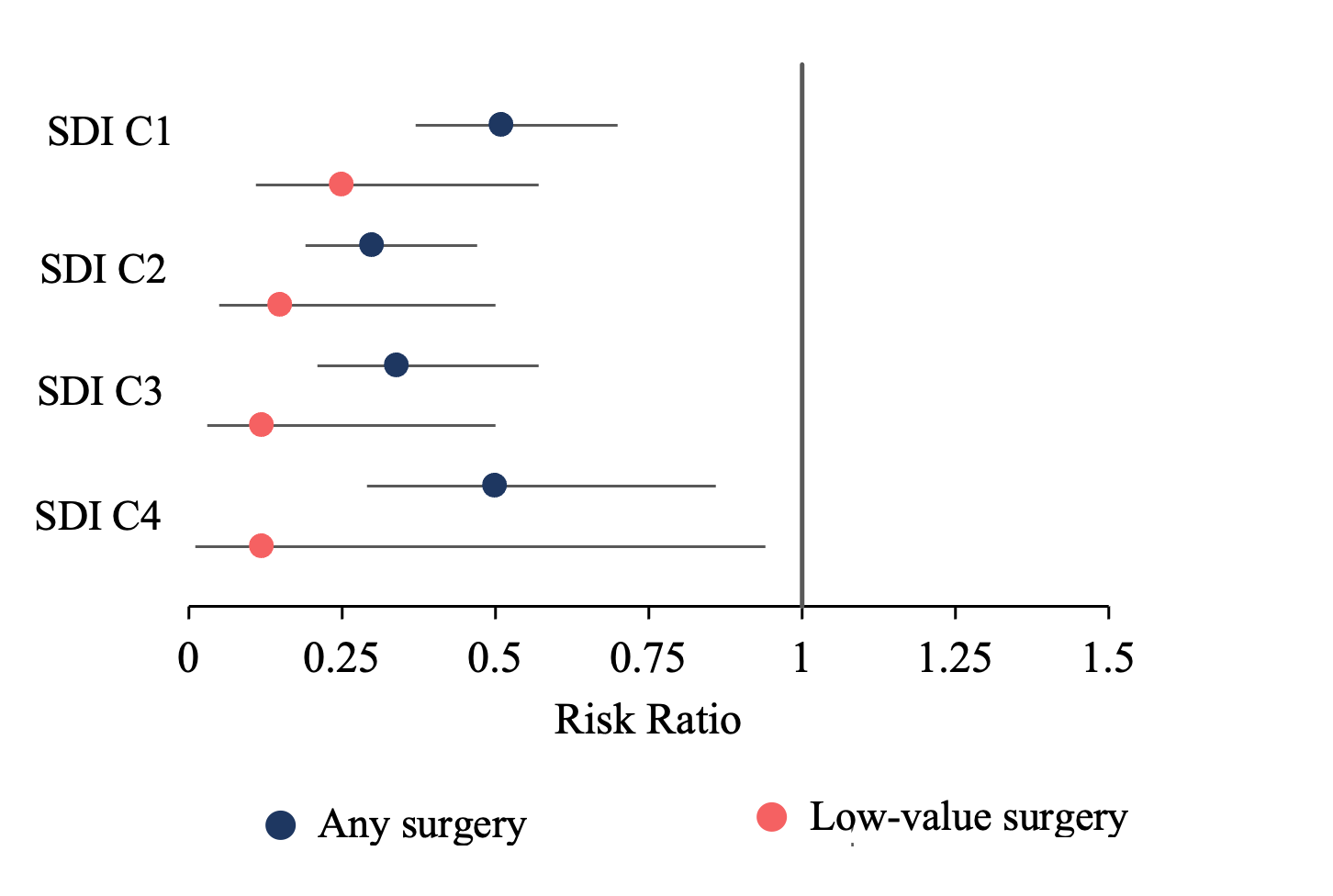

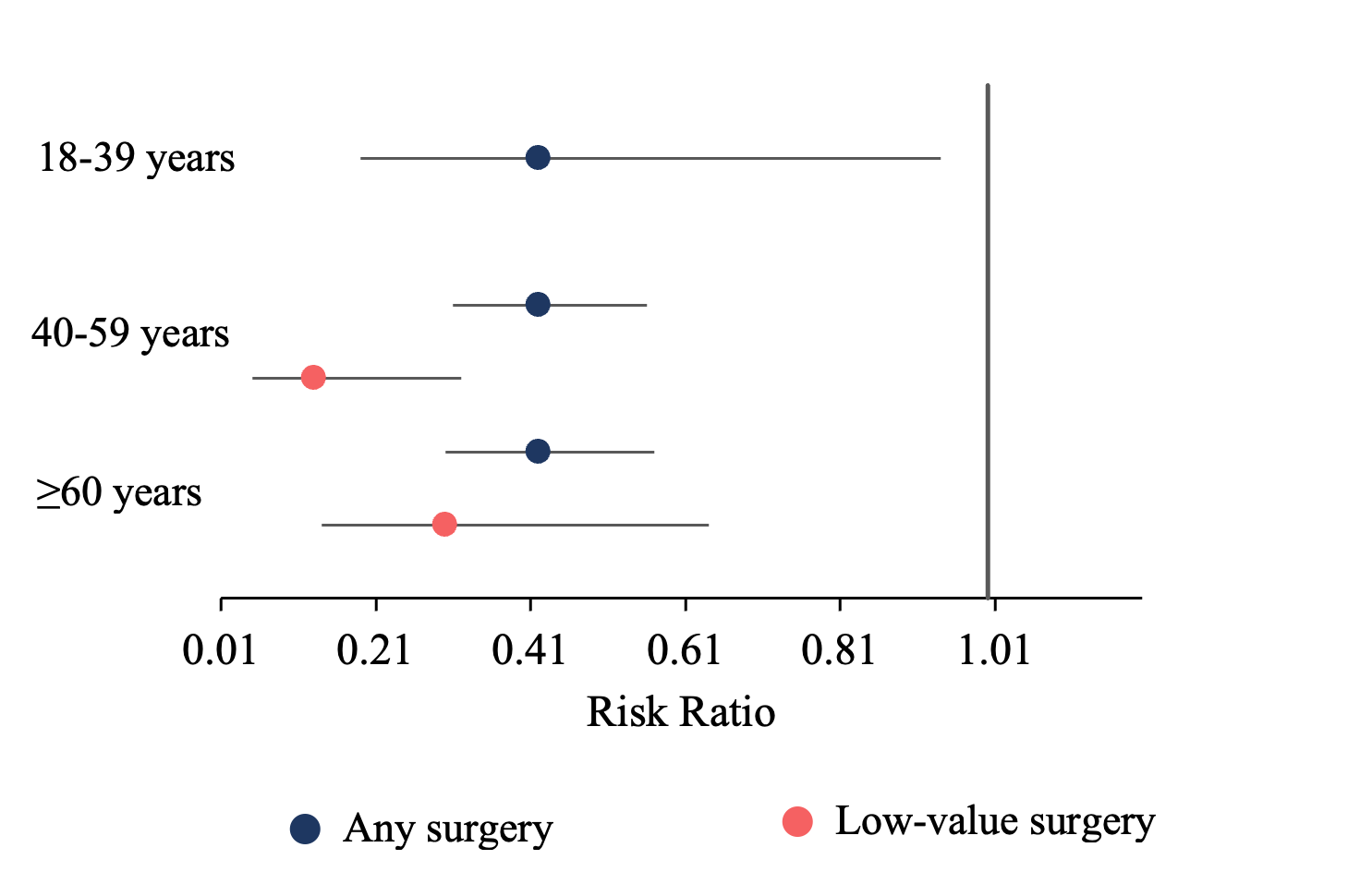


# Figure S3. Forest plots depicting the risk ratio for any surgery and low-value, stratified by (a) social deprivation index, and (b) age group. Dark blue dots represent any surgery; red dots represent low-value surgery. Error bars indicate 95% confidence intervals.

# Table S13. Latent-basis growth analysis (LBGA) estimates and model fit estimates: intention-to-treat approach.

| **Outcome** | **N** | **Intercept** | | **Program-end** | | **Mean change** | | **Model fit^1^** | | | |
| --- | --- | --- | --- | --- | --- | --- | --- | --- | --- | --- | --- |
|  |  | Mean (95%CI) | *P* | Mean (95%CI) | *P* | Mean (95%CI) | *P* | **Chi-sq, df \| *P*** | **CFI** | **RMSEA** | **SRMR** |
| **NPRS^2^** | 2,094 | 4.96  (4.87; 5.04) | <.001 | 2.75  (2.60; 2.88) | <.001 | -2.22  (-2.35; -2.08) | <.001 | 1 **\| 0.633** | **1.000** | **0.000** | **0.005** |
| **WPAI Activities^3^** | 2,094 | 29.86  (28.74; 30.99) | <.001 | 17.50  (16.09; 18.91) | <.001 | -12.36  (-13.93; -10.80) | <.001 | 1 **\| 0.238** | **1.000** | **0.016** | **0.009** |
| **WPAI Activities >0^4^** | 1,633 | 38.34  (37.20; 39.48) | <.001 | 20.22  (18.66; 21.79) | <.001 | -18.12  (-19.83; -16.41) | <.001 | 1 **\| 0.857** | **1.000** | **0.000** | **0.001** |
| **WPAI Overall^5^** | 1,813 | 19.44  (18.34; 20.53) | <.001 | 12.90  (11.47; 14.33) | <.001 | -6.54  (-7.98; -5.09) | <.001 | 1 **\| 0.716** | **1.000** | **0.000** | **0.022** |
| **WPAI Overall >0^6^** | 1,040 | 32.83  (31.47; 34.20) | <.001 | 18.58  (16.42; 20.74) | <.001 | -14.25  (-16.33; -12.18) | <.001 | 2 \| **0.576** | **1.000** | **0.000** | **0.017** |

#

Abbreviations: NPRS, Numerical Pain Rating Scale; WPAI, Work Productivity and Activity Impairment Questionnaire.

Note: **^1^**Model fitness was assessed through chi-squared test, root mean square error of approximation (RMSEA), confirmatory fit index (CFI), and standardized root mean square residual (SRMR), according to the criteria: chi-square *P*-value >0.05; CFI > 0.9; RMSEA < 0.05; and SRMR < 0.08. Values indicating good model fit are presented in bold.

^2^Across all timepoints, a total of 1,814 out of 6,282 expected data points were missing (28.9%). Statistical tests supported that missingness was completely at random (MCAR) (P = 0.113; R package MissMech).

^3^Across all timepoints, a total of 1,763 out of 6,282 expected data points were missing (28.1%). Statistical tests rejected MCAR (P < 0.001). Missingness was associated with observed variables (age, pain site, SDI score, and number of treatment weeks; P < 0.05), which is consistent with the assumption of missing at random (MAR).

^4^Across all timepoints, a total of 1,362 out of 4,899 expected data points were missing (27.8%). Statistical tests rejected MCAR (P < 0.001). Missingness was associated with observed variables (age, SDI score, pain site, and number of treatment weeks; P < 0.05), which is consistent with the assumption of MAR.

^5^Across all timepoints, a total of 2,325 out of 5,439 expected data points were missing (42.7%). Statistical tests rejected MCAR (P < 0.001). Missingness was associated with observed variables (gender, age, pain site, SDI score, baseline WPAI Overall, and number of treatment weeks; P < 0.05), which is consistent with the assumption of MAR.

^6^Across all timepoints, a total of 1,350 out of 3,120 expected data points were missing (43.3%). Statistical tests rejected MCAR (P < 0.001). Missingness was associated with observed variables (baseline WPAI Overall, age, and number of treatment weeks; P < 0.05), which is consistent with the assumption of MAR.

A marked reduction in pain severity of –2.22 points (95% CI -2.35; -2.08; *P*<.001) was observed by program-end, with 63.8% (N=1,037/1,626) of patients achieving clinically meaningful relief (Supplementary Table S8). Among employed patients (N=1,833), overall work productivity improved at program-end (mean change: –6.54 points, 95% CI –7.98; –5.09, *P*<.001), particularly for those reporting baseline impairment (mean change: –14.25 points, 95% CI –16.33; –12.18, *P*<.001). A significant reduction in daily activities limitation was also obtained (mean change: –12.36, 95% CI –13.93; –10.80, *P*<.001).

**References**

1. Benchimol, E.I., et al., *The REporting of studies Conducted using Observational Routinely-collected health Data (RECORD) statement.* PLoS Med, 2015. **12**(10): p. e1001885.

2. Motheral, B., et al., *A checklist for retrospective database studies--report of the ISPOR Task Force on Retrospective Databases.* Value Health, 2003. **6**(2): p. 90–7.

3. Tyree, P.T., B.K. Lind, and W.E. Lafferty, *Challenges of Using Medical Insurance Claims Data for Utilization Analysis.* Quality Assurance and Utilization Review, 2006. **21**(4): p. 269–275.

4. Wang, X., et al., *Meta-analysis of randomized trials comparing fusion surgery to non-surgical treatment for discogenic chronic low back pain.* J Back Musculoskelet Rehabil, 2015. **28**(4): p. 621–7.

5. Jacobs, W., et al., *Total disc replacement for chronic back pain in the presence of disc degeneration.* Cochrane Database Syst Rev, 2012(9): p. Cd008326.

6. Mo, Z., et al., *Exercise therapy versus surgery for lumbar spinal stenosis: A systematic review and meta-analysis.* Pak J Med Sci, 2018. **34**(4): p. 879–885.

7. Khan, M., et al., *Arthroscopic surgery for degenerative tears of the meniscus: a systematic review and meta-analysis.* Cmaj, 2014. **186**(14): p. 1057–64.

8. Sihvonen, R., et al., *Arthroscopic partial meniscectomy versus sham surgery for a degenerative meniscal tear.* N Engl J Med, 2013. **369**(26): p. 2515–24.

9. Abram, S.G.F., et al., *Arthroscopic partial meniscectomy for meniscal tears of the knee: a systematic review and meta-analysis.* Br J Sports Med, 2020. **54**(11): p. 652–663.

10. Lee, D.Y., et al., *Arthroscopic meniscal surgery versus conservative management in patients aged 40 years and older: a meta-analysis.* Arch Orthop Trauma Surg, 2018. **138**(12): p. 1731–1739.

11. Gwynne-Jones, J.H., et al., *The Outcomes of Nonoperative Management of Patients With Hip and Knee Osteoarthritis Triaged to a Physiotherapy-Led Clinic at Minimum 5-Year Follow-Up and Factors Associated With Progression to Surgery.* J Arthroplasty, 2020. **35**(6): p. 1497–1503.

12. Skou, S.T., et al., *The efficacy of 12 weeks non-surgical treatment for patients not eligible for total knee replacement: a randomized controlled trial with 1-year follow-up.* Osteoarthritis Cartilage, 2015. **23**(9): p. 1465–75.

13. Karjalainen, T.V., et al., *Surgery for rotator cuff tears.* Cochrane Database Syst Rev, 2019. **12**(12): p. Cd013502.

14. Schemitsch, C., et al., *Surgical repair versus conservative treatment and subacromial decompression for the treatment of rotator cuff tears: a meta-analysis of randomized trials.* Bone Joint J, 2019. **101-b**(9): p. 1100–1106.

15. Craig, R.S., et al., *Shoulder replacement surgery for osteoarthritis and rotator cuff tear arthropathy.* Cochrane Database of Systematic Reviews, 2020(4).

16. Martini, L., A. Velasquez, and A. Abache, *Non-Operative Treatment Options in Primary Glenohumeral Osteoarthritis: A Comprehensive Review.* Medical Research Archives, 2022. **10**(12).

17. Albishi W., M.K., Alaseem A., Awwad W., Alsanawi H., *The Effectiveness of Nonoperative Treatment Modalities in the Management of Frozen Shoulder: a Systematic Review of Randomized Controlled Trials.* Muscles Ligaments and Tendons Journal, 2022. **12**(02): p. 104:114.

18. Lähdeoja, T., et al., *Subacromial decompression surgery for adults with shoulder pain: a systematic review with meta-analysis.* Br J Sports Med, 2020. **54**(11): p. 665–673.

19. Karjalainen, T.V., et al., *Subacromial decompression surgery for rotator cuff disease.* Cochrane Database Syst Rev, 2019. **1**(1): p. Cd005619.

20. Nazari, G., et al., *The effectiveness of surgical vs conservative interventions on pain and function in patients with shoulder impingement syndrome. A systematic review and meta-analysis.* PLoS One, 2019. **14**(5): p. e0216961.

21. Familiari, F., et al., *Is acromioplasty necessary in the setting of full-thickness rotator cuff tears? A systematic review.* J Orthop Traumatol, 2015. **16**(3): p. 167–74.

22. Kavaja, L., et al., *Treatment after traumatic shoulder dislocation: a systematic review with a network meta-analysis.* Br J Sports Med, 2018. **52**(23): p. 1498–1506.

23. Dworkin, R.H., et al., *Interpreting the clinical importance of treatment outcomes in chronic pain clinical trials: IMMPACT recommendations.* J Pain, 2008. **9**(2): p. 105–21.

24. Ospina, M.B., et al., *A systematic review of measurement properties of instruments assessing presenteeism.* Am J Manag Care, 2015. **21**(2): p. e171–85.
